# Exploring the Autistic Brain: A Systematic Review of Diffusion Tensor Imaging Studies on Neural Connectivity in Autism Spectrum Disorder

**DOI:** 10.3390/brainsci15080824

**Published:** 2025-07-31

**Authors:** Giuseppe Marano, Georgios D. Kotzalidis, Maria Benedetta Anesini, Sara Barbonetti, Sara Rossi, Miriam Milintenda, Antonio Restaino, Mariateresa Acanfora, Gianandrea Traversi, Giorgio Veneziani, Maria Picilli, Tommaso Callovini, Carlo Lai, Eugenio Maria Mercuri, Gabriele Sani, Marianna Mazza

**Affiliations:** 1 Unit of Psychiatry, Fondazione Policlinico Universitario Agostino Gemelli, IRCCS, 00168 Rome, Italy; giorgio.kotzalidis@gmail.com (G.D.K.); mbenedetta@hotmail.it (M.B.A.); sara.barbonetti@gmail.com (S.B.); marianna.mazza@policlinicogemelli.it (M.M.); 2 Department of Neurosciences, Università Cattolica del Sacro Cuore, 00168 Rome, Italy; 3 Unit of Medical Genetics, Department of Laboratory Medicine, Ospedale Isola Tiberina-Gemelli Isola, 00186 Rome, Italy; gianandrea.traversi@gmail.com; 4 Department of Dynamic and Clinical Psychology, and Health Studies, Sapienza University of Rome, 00185 Rome, Italy; 5 Child Neurology and Psychiatry Unit, Fondazione Policlinico Universitario Agostino Gemelli, IRCCS, 00168 Rome, Italy; 6 Department Women Children and Public Health, Università Cattolica del Sacro Cuore, 00168 Rome, Italy

**Keywords:** autism spectrum disorder, diffusion tensor imaging, neuroimaging, white mater, radial diffusivity, axial diffusivity, fractional anisotropy, corpus callosum

## Abstract

**Background/Objectives**: Autism spectrum disorder (ASD) has been extensively studied through neuroimaging, primarily focusing on grey matter and more in children than in adults. Studies in children and adolescents fail to capture changes that may dampen with age, thus leaving only changes specific to ASD. While grey matter has been the primary focus, white matter (WM) may be more specific in identifying the particular biological signature of the neurodiversity of ASD. Diffusion tensor imaging (DTI) is the more appropriate tool to investigate WM in ASD. Despite being introduced in 1994, its application to ASD research began in 2001. Studies employing DTI identify altered fractional anisotropy (FA), mean diffusivity, and radial diffusivity (RD) in individuals with ASD compared to typically developing (TD) individuals. **Methods**: We systematically reviewed literature on 21 May 2025 on PubMed using the following strategy: (“autism spectrum”[ti] OR autistic[ti] OR ASD[ti] OR “high-functioning autism” OR Asperger*[ti] OR Rett*[ti]) AND (DTI[ti] OR “diffusion tensor”[ti] OR multimodal[ti] OR “white matter”[ti] OR tractograph*[ti]). Our search yielded 239 results, of which 26 were adult human studies and eligible. **Results**: Analysing the evidence, we obtained regionally diverse WM alterations in adult ASD, specifically in FA, MD, RD, axial diffusivity and kurtosis, neurite density, and orientation dispersion index, compared to TD individuals, mostly in frontal and interhemispheric tracts, association fibres, and subcortical projection pathways. These alterations were less prominent than those of children and adolescents, indicating that individuals with ASD may improve during brain maturation. **Conclusions**: Our findings suggest that white matter alterations in adults with ASD are regionally diverse but generally less pronounced than in younger populations. This may indicate a potential improvement or adaptation of brain structure during maturation. Further research is needed to clarify the neurobiological mechanisms underlying these changes and their implications for clinical outcomes.

## 1. Introduction

Autism spectrum disorder (ASD) encompasses a heterogeneous group of conditions related to brain development [1] characterised by persistent deficits in communication and social interaction as well as restricted and repetitive patterns of behaviour, interests, or activities that impair the individual’s optimal functioning [2,3,4]. The term ASD was coined in 2013 by the American Psychiatric Association [5] to encompass in one group terms like high-functioning autism, Asperger syndrome, Rett’s syndrome, and autism-related language and intellectual impairment. Currently, the 2017 prevalence of adult autism in the US is estimated to be approximately 2.21% [6]. The prevalence of autism has increased in the United States, from 1.1% in 2008 to 2.3% in 2018, with a male-to-female ratio of 4:1, and it affects about 2.3% of 8-year-olds and about 2.2% of adults [7,8,9]. It is not known whether the increased prevalence can be attributed to an actual increase in incidence or to an increased recognition by psychiatrists and other mental health professionals and/or changes in diagnostic criteria [10,11,12].

Autism started its career as a symptom in Eugen Bleuler’s newly coined term of schizophrenia [13]. The Swiss psychiatrist Bleuler subdivided schizophrenia symptoms into primary and secondary, with *Autismus* belonging to the latter ground (grund-) symptoms, and accessory symptoms. *Autismus* was one of the ground symptoms and was conceived as a closure in self. In fact, in his seminal 1911 book, page 52, Bleuler wrote “Die schwersten Schizophrenen, die gar keinen Verkehr mehr pflegen, leben in einer Welt für sich; sie haben sich mit ihren Wünschen, die sie als erfüllt betrachten, oder mit den Leiden ihrer Verfolgung in sich selbst verpuppt und beschränken den Kontakt mit der Außenwelt so weit als möglich. Diese Loslösung von der Wirklichkeit zusammen mit dem relativen und absoluten Überwiegen des Binnenlebens nennen wir Autismus”, i.e., “The most severe schizophrenics, who no longer have any social interaction, live in a world of their own; they have shelled themselves, with their desires, which they consider fulfilled, or with the suffering of their persecution, and limit contact with the outside world as much as possible. This detachment from reality, together with the relative and absolute predominance of inner life, is what we call autism”. After about 30 years, the concept of autism as we now conceive it was formulated by the Austrian psychiatrist Leo Kanner, who had emigrated to the US. He published his observations on eight boys and three girls [14], where he supported autism as a neurodevelopmental disorder. A similar view was developed earlier in the Soviet Union by the child neuropsychiatrist Grunya Efimovna Ssukhareva in 1926, who observed high-functioning children with what we would now call ASD [15], and which the Austrian child psychiatrist Hans Asperger [16], who observed relational difficulties in some of his patients, emulated. Uta Frith [17] and Simon Baron-Cohen [18] in the UK paved the way for further formulations of autism as a disorder involving lack of empathy and the inability to understand other people’s thoughts, i.e., the lack of “theory of mind”, a concept first investigated in non-human primates [19]. Authors converged in indicating mind-blindness as a core feature of autism [20]. The high-functioning vs. intellectually impaired dichotomy ended with the DSM-5 Task Force’s choice to adopt ASD for all types of autism [5], a decision that signalled a new start for this disorder. However, the idea of autism as a closure into one’s inner world, which was the basis of Eugen Bleuler’s definition, remained valid in the newer psychopathological formulations by Uta Frith and Sir Simon Baron-Cohen.

Research has identified links between ASD and several causes, with as yet not fully identified underpinnings [21,22,23]. Genetic factors are implicated in ASD, with the involvement of more than 800 genes, chromosomal deletions or duplications, and syndromes, although epigenetic, environmental, stochastic, and perinatal factors must be taken into account [23]. ASD is an early-onset disorder, and symptoms can be recognised by 12 to 24 months of age [24,25,26]. Some individuals, in whom there is no inadequate cognitive and language development, receive the diagnosis in adulthood, based on the disorders present at the time of clinical observation and developmental history [27,28].

Only a minority of people with ASD manage to live and work independently during adulthood, usually when there is low impairment of language and intellectual capacity; despite this, adult individuals use compensatory strategies to mask their social difficulties and are at a high risk of developing symptoms of anxiety and depression [29]. Regarding its course, stages of stabilisation or regression can be observed; however, ASD is not degenerative in nature [30,31,32]. Early diagnosis of autism is complex due to phenotypic and aetiological heterogeneity among individuals with ASD [33]. To date, no specific biomarkers have been identified for the diagnosis of ASD [8,34].

Voxel-based morphometry (VBM) is one of the most widely used methods to assess grey matter (GM) alterations. Through this method, volumetric abnormalities have been detected in various regions of the brain of individuals with ASD, including the fusiform gyri, hippocampus, amygdala, insula, frontal lobe, and cerebellum [35,36,37]. In adults, abnormal intra-regional variability in cortical thickness has been shown in many brain regions [38], with significantly increased grey matter volumes in mid-temporal, superior temporal, postcentral, and parahippocampal gyri. Grey matter volumes were reduced in the anterior cingulate cortex and the cerebellum in one meta-analysis [39], while another study showed increased grey matter volume in the anterior temporal and dorsolateral prefrontal regions and reduced volumes in occipital and medial parietal regions in ASD compared with controls [40]. Another study reported smaller frontal and temporal volumes in three adults with Rett’s syndrome [41], while other studies showed only trend differences [42,43]. These inconsistencies prevent us from thinking of VBM alterations as a reliable method of studying adult ASD. In summary, voxel-based morphometry studies have revealed heterogeneous alterations in grey matter volume in adults with ASD. In contrast, structural MRI analyses of white matter have not yielded consistent findings [44], underscoring the need for more specific techniques such as diffusion tensor imaging (DTI) to study white-matter microstructure.

The brain WM, which allows neurons and neuronal networks to communicate and function with high efficiency [45], is studied through DTI. This neuroimaging technique examines the integrity of WM, specifically through the movement of water molecules, producing three-dimensional images that help assess the microstructure of WM tracts. Fractional anisotropy (FA) is a quantitative measure derived from DTI that quantifies the degree of directional dependence (anisotropy) of water diffusion in a given region of the brain. Radial diffusivity (RD) measures the diffusion of water molecules perpendicular to fibre tracts, reflecting the myelination or integrity of axon membranes [46,47,48]. Another diffusivity measure is mean diffusivity (MD). Other DTI measures include axial kurtosis (AK), axial diffusivity (AD), and the orientation dispersion index (ODI).

Although diffusion tensor imaging (DTI) was first proposed as a technique in 1994 [49], studies employing this technique in autism were not performed until 2001 [50]. Brain development in individuals with ASD follows a different trajectory than in neurotypical (NT) individuals [51,52] (we will here refer to NT individuals and typically developing (TD) individuals interchangeably and treat the terms like synonyms). Unlike TD children, the brain development of children with ASD does not follow a uniform pattern [53,54,55]. While developmental brain trajectories in individuals with ASD vary, with some showing convergence with typical patterns during adulthood and others maintaining divergent profiles, this neurobiological heterogeneity does not explain the reported decline in prevalence from childhood to adulthood, which is more likely attributable to differences in diagnostic criteria, symptom masking, and service access [56]. Focusing on WM alterations in ASD during the period of continuous brain development does not capture the end results of brain maturation, which occur at about 25 years of age [57]. We believe WM alterations in adult ASD better reflect autism’s biological signature than other biological markers. We thus decided to focus on WM alterations in ASD in adult individuals with ASD. The aim of this review was to analyse specific patterns of altered DTI in adults, based on the existing literature.

## 2. Materials and Methods

To obtain the literature on DTI in living ASD individuals, we investigated the PubMed database because it gathers all relevant peer-reviewed literature and is simple and straightforward to investigate. On 21 May 2025, we used the following strategy: (“autism spectrum”[ti] OR autistic[ti] OR ASD[ti] OR “high-functioning autism” OR Asperger*[ti] OR Rett*[ti]) AND (DTI[ti] OR “diffusion tensor”[ti] OR multimodal[ti] OR “white matter”[ti] OR tractograph*[ti]). Limiting search keywords to titles had the purpose of avoiding generic literature that lumped other disorders with ASD and increasing the specificity of the search. Using tractography/tractographic and multimodal among keywords aimed to avoid missing studies with DTI data, as works with such words in their title often provide DTI data. WM structure is usually investigated in current times in vivo using DTI. However, recently, multimodal approaches have been increasingly adopted to study the autistic brain. These combine several neuroimaging techniques (and even tasks), including [58,59,60] or not including DTI [61,62,63]. Hence, in our search strategy, we included as a search term the word “multimodal”, so as to capture as many DTI studies as possible, including those studies that provided DTI measures separately. We further added the PsycINFO and CINAHL databases, adapting the strategy to TI (autism spectrum OR autistic OR ASD OR high-functioning autism OR Asperger OR Rett) AND TI (DTI OR diffusion tensor OR tractography) AND TI (adult OR adults).

We conducted our systematic review following the Preferred Reporting Items for Systematic Reviews and Meta-Analyses (PRISMA) 2020 Statement [64].

### Eligibility Criteria

We included studies reporting DTI data for adult individuals with ASD. When studies focused on mixed populations, i.e., children/adolescents and adults, they were included only if they reported adult data separately. When data from adult individuals were pooled with those of children and adolescents, we excluded them and labelled them “Pooled”. Excluded were studies involving children and adolescents (collectively labelled “Children”); studies not reporting on DTI (labelled “No DTI”); reviews, consensus conferences, guidelines and meta-analyses (collectively labelled as “Review”); studies not reporting data from ASD individuals (labelled “No ASD”); those lumping ASD individuals with those with other diagnoses in analysing their data (termed “Lumping”); studies reporting on the same or overlapping samples of individuals included in the study (labelled “Overlap”); studies with a design unsuitable to provide DTI data from adult ASD individuals (labelled “Unfocused”); protocols with no data (labelled “Protocol”); animal/in vitro studies (labelled “Animal”), post-mortem studies (labelled “Post-mortem”), as they could not provide data on living people; case reports and case series (labelled as “Case”); and editorials, letters to the editor, position papers, and viewpoints, collectively labelled “Opinion”. We also excluded all studies that emerged serendipitously from the search without having any relationship with the subject matter (labelled as “Unrelated”). We also eliminated duplicates (same article emerging more than once or corrections to already emerged articles, labelled as “Duplicate”), and “Retracted” articles (by authors or editors/publishers). The exclusion categories and reasons for excluding each study appear in the Supplement (Supplementary Table S1). We also excluded studies not subjected to peer review, such as conference abstracts with insufficient data, as well as grey literature.

All authors independently performed the PubMed search after consensus on the search strategy and completed the screening based on all retrieved articles. All authors participated in Delphi rounds to establish eligibility in agreement with the inclusion criteria. Disagreements were solved through discussion and consensus involving all authors. Two rounds were sufficient to obtain consensus.

Eligible studies were searched for DTI measures and are summarised in Table 1. The main outcomes of these studies were terms related to DTI, i.e., FA, RD, MD, AK, AD, and ODI.

We controlled the risk-of-bias/quality of eligible studies through the Quality Assessment with Diverse Studies (QuADS) tool [65]. This tool proved to be valid in analysing DTI data [66]. We performed an evaluation of the risk-of-bias/quality for each included study. The results are shown in the Online Supplement (Supplementary Table S2). We registered our review on the Open Science Framework (OSF) platform with the identifier https://doi.org/10.17605/OSF.IO/SKCQ5.

Although several of the included studies shared similar designs and reported comparable DTI metrics such as FA, RD, and MD, a formal quantitative meta-analysis was not feasible due to several critical limitations. In fact, many studies lacked sufficient statistical detail. Essential data such as group-level means, standard deviations, t-values, or standardised effect sizes were often missing, preventing the reliable calculation of pooled metrics across studies. There was also considerable methodological heterogeneity among the included studies. Differences were observed in the following: imaging models, including not only classical DTI but also advanced methods like diffusion kurtosis imaging (DKI) and neurite orientation dispersion and density imaging (NODDI), the latter of which provides more detailed microstructural information by estimating neurite density and orientation dispersion; data analysis pipelines, with some studies using tract-based spatial statistics (TBSS), a voxelwise analysis method projecting individual FA maps onto a group mean white-matter skeleton, while others used region-of-interest (ROI)-based analyses, tractography, or whole-brain approaches; anatomical targets, which varied widely (e.g., corpus callosum, superior longitudinal fasciculus, uncinate fasciculus); and participant characteristics, including age ranges, sex distribution, intellectual functioning, and psychiatric or neurodevelopmental comorbidities.

Finally, in several studies, the relevant results were presented only as figures, plots, or narrative summaries, without the underlying numeric data needed for meta-analytic computation. Given these limitations, a quantitative synthesis would risk introducing misleading conclusions based on incomplete or non-comparable data. We therefore opted for a systematic qualitative synthesis, which allowed us to identify consistent and regionally specific patterns of white-matter microstructural alterations in adults with ASD. This approach, while descriptive, still provides a valuable integrative perspective on the current state of the literature and its clinical implications.

**Table 1:** Summary of included studies in chronological order.

Study	Population Studied	Technique	Design/Tools	Results	Conclusion/Observations
Bloemen et al., 2010 [67]	13 ♂ Asperger syndrome ($\bar{x}$ age 39.00 ± 9.70 yrs, range = 23–54, IQ 110 ± 15.7, range 88–133) vs. 13 ♂ TD ($\bar{x}$ age 37.00 ± 9.60 yrs, range = 25–52, IQ 115 ± 14.4, range 89–133).	DTI, GE Signa 1.5T LX system, with actively shielded magnetic field gradients, max. amplitude 40 mT^−1^.	Cross-sectional, WAIS→inclusion, MRI acquisition, calculation of initial FA, MD, and RD images, normalised through SPM2; images averaged and smoothed. Permutation 1000 for voxels and clusters; localisation in MNI space. Assessment through ICD-10 criteria and FSIQ.	↓ FA in Asperger vs. TD in CC, IFOF, ILF in IFG, cuneus, frontal lobe, temporal lobe, ATR in MFG and CC, UF in MFG and CC, SLF in cingulate, and cortico-spinal tract in CC and parietal lobe; ↑ RD in Asperger vs. TD in 16 clusters and ↓ RD in two (cerebellum and CC); ↓ RD in Asperger vs. TD in the brainstem.	Results are preliminary due to small sample size; results in table reported ↑ FA, while actually there was a ↓ in the reported areas and tracts. Results did not depend on age, IQ, or instruments used to assess ASD. Despite this, the study showed WM differences in ASD. Study only on ♂.
Thomas et al., 2011 [68]	12 ♂ high-functioning ASD ($\bar{x}$ age 28.50 ± 9.80 yrs, range = 19–49, IQ 106.92 ± 10.47, range 95–126) vs. 18 ♂ TD matched by age, gender, handedness, and IQ ($\bar{x}$ age 33.40 ± 4.10 yrs; IQ 106.91 ± 9.91).	3T MRI (Siemens), T1-weighted 3-D MPRAGE. DTIstudio to calculate FA→smoothing.	Cross-sectional, WAIS. Assessment through ADI-R, ADOS-G. For DTI, extended ROI approach. Tractography used to investigate fibre integrity in Fma, Fmi, body of CC, ILF, IFOF, and UF.	↓ Macro-structural integrity of fibres in CC in high-functioning ASD vs. TD; Tukey post-hoc analysis showed ↓ numbers of streamlines and voxels in FMi; no alterations in FMa. Leftward asymmetry for streamlines in the high-functioning ASD group for ILF, IFOF, and UF; ↓ numbers of streamlines in these tracts correlated with ↑ scores on ADI-R. No gross alterations in structural integrity between the two groups.	Small sample size; the major inter-hemispheric and intra-hemispheric visual-association tracts are altered in individuals with high-functioning ASD, although not as much as was anticipated. Study only on ♂.
Bakhtiari et al., 2012 [69]	16, 15 ♂ 1 ♀ high-functioning ASD adolescents (≤20 yrs of age) ($\bar{x}$ age 15.5 ± 2.8 yrs, IQ 108.1 ± 13.5) vs. 18, 17 ♂ 1 ♀ TD adolescents ($\bar{x}$ age 15.5 ± 2.0 yrs, IQ 111.8 ± 13.7) vs. 14, 12 ♂ 2 ♀ adult high-functioning ASD (>20 yrs of age) ($\bar{x}$ age 28.1 ± 6.5 yrs, IQ 110.3 ± 15.8) vs. 19, 16 ♂ 3 ♀ adult TD ($\bar{x}$ age 8.6 ± 5.6 yrs, IQ 112.3 ± 8.5).	3T high-speed echoplanar-MRI (Siemens). Head stabilisation through foam padding. Diffusion-weighted data with 70 directions. DTI using FSL.	Cross-sectional, AQ, ADOS-2, ADI-R; DTI spaces in MNI. Sought interactions group × age to define differences between ASD and TD, and correlations between FA and each of AQ (entire population), ADOS-2, and ADI-R (entire population and groups).	↓ FA bilaterally in “adolescent” ASD vs. TD in IFOF, ILF, SLF, ATR, UF, cortico-spinal tract, cingulum, FMi, CC, FMa. No differences in FA between adult ASD and TD. FA correlated positively with age. In adults with ASD, IFOF FA values negatively correlated with ADOS-2 communication scores and with ADOS-2 and ADI-R social scores, ILF FA with ADOS-2 social scores, and AF in CC splenium with ADI-R communication scores.	The >20 yr age cutoff for adulthood is arbitrary. ↓ FA in “adolescents” with ASD was no longer present in adult ASD individuals, indicating a convergence with “typicality” with increasing age. However, sample sizes were small.
Kleinhans et al., 2012 [70]	28 participants with ASD (16 ♂, 9 ♀; $\bar{x}$ age 21.29 ± 5.66 yrs, range = 13.72–35.59) and 33 TD controls (22 ♂, 6♀; $\bar{x}$ age 21.31 ± 7.269 yrs, range = 13.58–40.92) matched for age and IQ. ASD DSM-IV diagnoses confirmed with ADI-R and ADOS-2. Data from 5 TD and 3 ASD participants excluded due to incidental MRI finding, clinically significant elevation ↑ social anxiety, excessive artefacts.	3T MRI. T1-weighted MPRAGE. DTI with 32 gradient directions.	A cross-sectional study was conducted to determine whether WM abnormalities, as measured by DTI, were present in high-functioning participants with ASD and whether age-related changes in WM microstructure differed between participants with ASD and TD. ADI-R and ADOS questionnaires were used to confirm the diagnosis of ASD.	The ASD group had widespread ↓ in WM integrity, characterised by ↓ FA, ↑ RD and MD across major WM tracts, including association fibres, projection fibres, commissural fibres, and brainstem tracts. No significant group differences were found in AxD, suggesting that these abnormalities are likely related to myelin dysfunction rather than axonal degeneration. FA values typically ↓ with age in TD individuals; the ASD group exhibited age-related ↑ in FA and ↓ in RD and MD, suggesting a potential compensatory normalisation of WM microstructure in early adulthood.	A key aspect was the analysis of the age-by-diagnosis interaction, which revealed that WM abnormalities in ASD follow an atypical developmental trajectory. While differences from TD are more pronounced during adolescence, certain WM parameters appear to partially normalise in adulthood, suggesting potential compensatory mechanisms or delayed maturation. However, abnormalities persist, indicating that disrupted brain connectivity remains a stable feature of ASD.
Mueller et al., 2013 [71]	12 adults with high-functioning ASD (9 ♂, 3 ♀; $\bar{x}$ age 35.5 ± 11.4 yrs) and 12 NT (8 ♂, 4 ♀; $\bar{x}$ age 33.3 ± 9.0 yrs). All participants with full-scale IQ > 85. ASD diagnosed according to ICD-10, and autistic traits assessed with AQ; excluded participants with major psychiatric, neurological, genetic, metabolic, and infectious disorders/diseases.	3T MRI. T1-weighted MPRAGE, DTI analysed using TBSS. VBM and resting-state fcMRI performed.	Cross-sectional study comparing structural and functional brain imaging between adults with high-functioning ASD and matched NT using multimodal MRI (DTI, VBM, fcMRI) to investigate common alterations in WM and GM. Psychological tests, including FPI and QEAS, used to correlate with the most significant findings. Assessment with AQ and FSIQ.	Widespread ↓ WM microstructural integrity in adults with high-functioning ASD compared to NT. ↓ FA in three main clusters: a right-lateralised cluster extending from CC splenium into the SLF, within the parietal and temporal lobes, and into RLOC; a left anterior cluster involving the anterior CC, ACC, and MFG; and bilateral corticospinal tract. ↓ FA in the right TPJ area overlapped with ↓ resting-state functional connectivity within the DAN, while the left frontal cluster co-localised with ↓ resting-state functional connectivity in the LFPN. Finally, FA values in the right TPJ area showed a significant positive correlation with emotionality scores on the FPI (*r* = 0.74, corrected *p* < 0.05).	Convergent sites of structural and functional alterations in higher-order association cortex areas suggest higher-order multisensory integration. FA in the right TPJ area correlated with ↓ emotionality, linking WM abnormalities to socio-emotional traits in high-functioning ASD.
Roine et al., 2013 [72] *	14 ♂ adult ASD participants ≤40-yrs-old ($\bar{x}$ age 28.6 ± 5.7 yrs; IQ 125.1 ± 14.5) and 19 ♂ gender-, age-, and QI-matched TD ≤40-yrs-old ($\bar{x}$ age 26.4 ± 4.7 yrs; IQ 127.9 ± 10.0). ASD diagnosed according to the ICD-10.	Signa VH/i 3T scanner for MRI acquisition. Spin-echo pulsed sequence of 60 unique gradient orientations × 2. TBSS and CSD *.	Cross-sectional. AQ, EQ, SQ, Eyes Test, FRT. FA and MD images introduced in TBSS. FA data analysed according to skeletonisation, tractography, and TBSS-CSD.	↑ AQ and EQ, but not SQ scores in ASD vs. TD; ↓ FA, but not MD and CP in ASD vs. TD. FA correlated positively with AQ scores. FA data were stronger in skeleton than in tractography. ↑ FA in ASD vs. TD in the temporal part of the SLF, corticospinal tract, CC splenium, ATR, inferior IFOF, PTR, UF and ILF *. ↑ FA in ASD vs. TD with CSD in left ILF *.	Small men-only samples, no use of ADOS-2, and ADI-R limit generalisability and strength of results. ↑ and ↓ FA in ASD vs. TD according to areas. Results indicate limited structural WM integrity impairment in ASD.
Peeva et al., 2013 [73]	18 participants with ASD (15 ♂, 3 ♀, $\bar{x}$ age 25.6 ± 9.2; age range 16–50 yrs) and 18 NT participants (12 ♂, 6 ♀, $\bar{x}$ age 28.5 ± 8.7; age range = 19–44 yrs). ASD diagnoses confirmed using ADI-R and ADOS-4 tools. Excluded participants with DSM-IV comorbid psychiatric conditions, substance use disorders, or autism-related medical conditions.	3T MRI. T1-weighted MPRAGE for anatomical registration and cortical parcellation. DTI with 72 gradient directions. FMRIB Diffusion Toolbox (FDT).	Study is observational and employs a case-control design. Specifically, it investigates the integrity of WM projections in the speech-production networks of high-functioning individuals with ASD in order to identify potential causes of ASD-related speech impairments at the neuroanatomical level.	Highlights ↓ WM connectivity between the left vPMC and the left SMA in individuals with high-functioning ASD compared to NT controls. This structural alteration may affect the initiation and motor control of speech, suggesting a potential deficit in the neural mechanisms supporting speech production. No significant differences were found in other examined WM tracts or in measures of FA or tract volume. Additionally, impaired mirror neuron activity may result from the disrupted influence of the SMA on the vPMC.	Supports the idea that autism is a disorder of brain connectivity, in which structural abnormalities in WM can affect fundamental aspects of language and communication. Moreover, since the connectivity deficit between the vPMC and SMA is also present in ASD individuals with normal language abilities, targeted interventions aimed at enhancing brain connectivity could improve speech production.
Itahashi et al. 2015 [59]	46 adult ♂ with ASD from Karasuyama Hospital outpatient units, along with 46 age-matched NT ♂ controls recruited through advertisements. ASD diagnoses were based on DSM-IV criteria and a review of medical records. IQ scores were assessed using the WAIS-III or WAIS-R for participants with ASD and the JART for those without ASD. Participants completed the Japanese version of the AQ test. None met diagnostic criteria for any psychiatric disorder or had a medical or neurological history.	1.5T MRI. T1-weighted SPGR 3D sequence for anatomical registration and cortical parcellation. DWI with 30 gradient directions. FDT and TBSS to assess WM connectivity.	A multimodal neuroimaging cross-sectional study employing structural MRI and DTI to investigate alterations in grey and WM morphology in adults with ASD. Using LICA, the study integrates structural and connectivity data to identify shared patterns of neuroanatomical abnormalities.	Identified significant morphological alterations in GM and WM in individuals with high-functioning ASD compared to controls. Specifically, **↓** in GM volume were observed in the bilateral fusiform gyri, bilateral orbitofrontal cortices, and bilateral pre- and post-central gyri. Volumetric **↑** were detected in the anterior temporal poles and putamen. Additionally, WM analysis revealed **↓** FA in multiple major tracts, including the bilateral inferior longitudinal fasciculi and bilateral corticospinal tracts. A significantly **↑** MD for ASD was found in projection, commissural, and association fibres.	Findings suggest disruptions in neural networks responsible for cognitive and affective functions in ASD. Results provide a more comprehensive view of ASD neuroanatomy and indicate directions for future research for understanding the pathological mechanisms underlying these structural alterations. The study emphasises the importance of developing multimodal data-fusion approaches to identify new correlations between neuroanatomical abnormalities and cognitive-behavioural deficits in ASD. They also point to further studies focusing on female participants.
Libero et al., 2015 [60]	19 high-functioning adults with ASD (15 ♂, 4 ♀; $\bar{x}$ age: 27.1 yrs) and 18 TD peers (14 ♂, 4 ♀; $\bar{x}$ age: 24.6 years). ASD diagnoses were confirmed using ADI-R and ADOS tools. TD participants screened through a self-report history questionnaire to rule out neurological disorders. FSIQ, VIQ, and PIQ were measured using the WASI, handedness was evaluated with the EHI, and ASD symptoms were assessed using RAADS-R.	3T MRI with high-resolution T1-weighted MPRAGE, DTI with 46 gradient directions, and ^1^H-MRS using a PRESS sequence targeting the dACC and PCC; mrDiffusion for DTI, FreeSurfer for cortical segmentation, and SPM8 for VBM analysis.	Observational, cross-sectional study using a multimodal neuroimaging approach to classify ASD. It integrates structural MRI, DTI, and ^1^H-MRS within the same cohort.	Findings revealed ↑ cortical thickness in the left cingulate cortex, left inferior frontal gyrus, left inferior temporal cortex, and right precuneus, alongside ↓ cortical thickness in the right cuneus and precentral gyrus. DTI analyses indicated ↓ FA and ↑ RD in the forceps minor of the CC, suggesting altered WM connectivity. Additionally, ^1^H-MRS detected a ↓ in the NAA/Cr ratio in the dorsal anterior cingulate cortex, indicative of neuronal dysfunction.	The combination of structural, diffusion-based, and neurochemical markers proved to be a more reliable diagnostic tool than single-modality approaches, suggesting that ASD-related brain alterations span different levels of neural organisation. Future studies should focus on larger, more diverse samples, including younger and lower-functioning individuals, to improve the generalizability of these findings.
Kirkovski et al., 2015 [74]	25 adults with high-functioning ASD (12 ♂, 13 ♀) and 24 age-, sex- and IQ-matched NT controls (12 ♂, 12 ♀), FSIQ ≥ 85.	3T MRI; high-resolution MPRAGE T1-weighted structural MRI for anatomical imaging. DWI with dual-spin-echo EPI sequence to analyse WM integrity.	Cross-sectional study comparing DTI metrics (FA, MD, RD, and AD) between the ASD group and the control group. Assessment with RAADS-R.	No significant differences in FA, MD, RD, or AD between adults with high-functioning ASD and NT controls. No effects of biological sex on these measures.	No differences in WM microstructure between adult ASD population and NT individuals.
Ecker et al., 2016 [75]	51 ♂ adults with ASD ($\bar{x}$ age 26 ± 7 yrs, range 18–43 yrs) and 48 NT ♂ ($\bar{x}$ age 28 ± 6 yrs, TD range 18–43 yrs).	3T. Structural MRI (T1-weighted imaging). DTI with spin-echo EPI sequence.	Cross-sectional study investigating the relationship between cortical gyrification and WM connectivity by comparing lGI and DTI metrics between the ASD and control groups. Assessment through ICD-10 and ADI-R.	ASD groups showed increased gyrification in the left pre- and post-CG. Corresponding WM tracts showed increased AD, particularly in fibres near the cortical surface. Significant correlation between elevated lGI and increased AD in short tracts.	Differences in GM neuroanatomy and WM connectivity in individuals with ASD are linked and may be characterised by common aetiological pathways.
Libero et al., 2016 [76]	42 high-functioning children, adolescents, and adults with ASD (36 ♂ and 6 ♀, $\bar{x}$ age 19.9 yrs) and 44 TD (37 ♂ and 7 ♀, $\bar{x}$ age 20.01 yrs with no psychiatric/neurological disorders such as ASD, ADHD, or Tourette’s disorder), matched according to age and IQ. ASD and TD groups did not differ on age, VIQ, PIQ, or FSIQ. Clinical diagnosis of ASD based on ADI-R and ADOS-G.	3T. Structural MRI (T1-weighted imaging). DTI with a spin-echo EPI sequence. Data analysed using automated fibre quantification, which provides the diffusion profile of an entire WM tract and avoids voxel misalignment errors.	Cross-sectional study to determine WM abnormalities through DTI. Psychometric tools included AQ and RAADS-R. VIQ, PIQ, and FSIQ were assessed using the WASI and EHI.	The ASD group showed significant ↓ FA in anterior L-SLF and significant ↑RD in L-SLF. ↑ FA in the anterior L-SLF with age in all participants. The correlation between FA for the L-SLF and RAADS-R scores in adult participants and AQ in child participants was not significant.	↓ FA in the anterior L-SLF may suggest alterations in connectivity with the frontal lobe, to which it is connected. No significant differences were found in the cingulum bundle, inferior longitudinal fasciculus, and CC.
Nickel et al., 2017 [77]	30 high-functioning adults with ASD (19 ♂ and 11 ♀, $\bar{x}$ age 35.40 ± 9.065 yrs, IQ > 100) and 30 TD (19 ♂ and 11 ♀, $\bar{x}$ age 35.53 ± 8.303 yrs, with no psychiatric/neurological history), matched according to age, gender, and IQ. Clinical diagnosis of ASD based on ICD-10 and DSM-IV.	3T MRI, MPRAGE T1-weighted anatomical scan. DTI with 61 spatial directions.	Cross-sectional study to determine WM abnormalities through DTI. Psychometric tools included AQ, EQ, SRS, BVAQ, AAA, and BDI. ADI-R and ADOS-G were used in unclear cases.	The ASD group showed significant ↓ FA in the genu and the body of the CC and significant ↑ MD in the sACC. No significant correlations between MD, FA, and autistic symptom load according to AQ and EQ.	A ↓ FA within the genu of the CC indicates reduced directionality of diffusion, suggesting diminished interhemispheric fibres; this may explain the functional deficits observed in the frontal lobe with which the CC is connected. ↑ MD in the sACC correlates with previously observed ACC alterations, potentially linking to theory-of-mind deficits and atypical von Economo neuronal development. These neurones facilitate rapid intuitive assessment, and their dysfunction may contribute to social impairments in ASD.
Yamagata et al., 2018 [78]	60 participants (60 ♂, IQ ≥ 80) consisting of 30 pairs of biological siblings; 15 pairs discordant for ASD (1 prtc with ADHD, 1 prtc with DD), 15 pairs TD; ASD diagnosis based on DSM-IV-TR and ADOS-2, MINI used to confirm absence of ASD in unaffected sibling. Handedness evaluated using EHI, IQ assessed via WAIS-III or WAIS-R. All participants completed AQ-J.	3T MRI. DTI; EPI spin-echo sequence	Cross-sectional study, investigated WM differences in ASD-discordant siblings using TD pairs to control variance. DTI data were analysed using TBSS and ROI-based approaches. Paired *t*-tests were applied for WM differences, ICC assessed structural similarity, and bootstrapping used to evaluate DTI metric variations. Pre-processing and analyses were performed using FSL. ANOVA was used to examine AQ-J variances.	No significant differences in age, full IQ, verbal IQ, performance IQ, or handedness between ASD participants and their unaffected siblings. AQ total and subscale scores ↑ in ASD, while ADI-R scores ↓ in unaffected siblings. No significant differences in full IQ, verbal IQ, performance IQ, handedness, or AQ scores in TD pairs. Unaffected siblings showed ↑ social skills subscale scores compared to all participants. No significant differences in any DTI parameters between ASD participants and their unaffected siblings. Significant RD similarity in LST and marginal FA correlation among TD siblings. A marginally significant AD correlation was observed in RUF. In ASD participants and their unaffected siblings, significant ICCs for FA and RD were found in MCP, with marginal ICCs in RALIC and PCT. A significantly large difference in AD was observed in LST between ASD participants and their unaffected siblings.	This study introduced a novel approach to distinguish neural correlates of ASD diagnosis from neuro-endophenotypes by including both ASD-discordant and TD sibling pairs. RD similarity in LST suggests a genetic contribution, while AD differences may be linked to ASD diagnosis in individuals with an ASD endophenotype.
Hattori et al., 2019 [79]	30 r-h participants:15 ASD (7 ♂, 8 ♀; $\bar{x}$ age 39.8 ± 7.9 yr(s)) diagnosed via DSM-5, 15 NT (10 ♂, 5 ♀; $\bar{x}$ age 38.6 ± 7.9 yr(s)). AQ, SQ, and EQ used to assess autism traits.	3T MRI, DKI; multishell DWI with 32 diffusion directions; EPI sequence; FA, MD, AD, RD, MK, RK, and AK.	Cross-sectional study comparing WM between ASD and NT. TBSS and ROI analyses (GLM in FSL, 5000 permutations) used to assess WM differences. Spearman’s correlation tested AQ-J, SQ relation to AK.	ASD group showed ↓ AK in splenium and body of CC vs. TD. No significant differences in FA, MD, RD, or MK. AK negatively correlated with AQ-J and SQ in multiple WM tracts (e.g., UF, IFOF, ILF, SLF).	DKI detected WM microstructural alterations in ASD undetected by DTI. AK reduction in CC may reflect axonal deficits in ASD. AK correlation with AQ-J, SQ suggests potential marker for ASD severity.
Yassin et al., 2019 [80]	76 r-h ♂ participants: 39 ASD (high-functioning ASD, $\bar{x}$ age 30.1 ± 6.8 yrs, IQ > 80), 37 TD ($\bar{x}$ age 31.5 ± 4.6 yrs, IQ > 80). ASD diagnosed via DSM-5. PA/MA recorded.	DTI on a 3T MRI (GE Signa HDxt, SW v14.0); single-shot spin-echo EPI sequence, 30 directions, 2.4 mm slices, FOV 240 × 240 mm, acquisition time 12:20 min).	Cross-sectional study analysing WM using DTI. TBSS and ROI analyses assessed WM differences; regression models evaluated PA and MA effects; multiple linear regression for MD/RD prediction.	↑ MD and RD in ASD (IFOF, R-ILF, SLF, UF, CG, forceps minor, ATR, and R-CST); no FA or AD differences. PA positively correlated with MD and RD in affected WM tracts; MA showed no effect.	This study supports PA as a factor influencing WM disparities in ASD, particularly with increased MD and RD in major tracts. The observed WM alterations suggest a dysmyelination process rather than axonal degeneration, reinforcing the hypothesis of a neurodevelopmental mechanism related to parental age.
Mohajer et al., 2019 [81]	52 r-h ♂ participants: 26 high-functioning ASD ($\bar{x}$ age 36.7 ± 16.1 yrs), 26 TD ($\bar{x}$ age 37.5 ± 14.3 yr(s)). ASD diagnosed via ADOS-2. BDI-II used to assess depressive symptoms.	DTI on a 3T MRI; 32 diffusion directions. Explore DTI toolbox used for pre-processing; FA and MD analysed via Mori WM atlas.	Cross-sectional study assessing WM integrity in ASD with comorbid depressive symptoms. Linear regression models evaluated BDI relation to FA and MD. FDR correction applied to multiple comparisons.	↓ FA and ↑ MD in ASD with depressive symptoms, mainly in bilateral ALIC and corona radiata. Significant FA-BDI association in LALIC, with weaker effects in external capsules and UF.	This study highlights that WM alterations in ASD with depressive symptoms mirror those found in TD individuals with depression, indicating shared neurobiological substrates between these conditions. Findings suggest shared neurobiological substrates for ASD-related and TD depression.
Haigh et al., 2020 [82]	45 participants with ASD, age 17–45 yrs; 36 ♂ and 9 ♀) and 20 TD, age 18–41 yrs; (36 ♂ and 9 ♀) matched on age, gender, IQ, race, and employment status.	3T MRI, DTI, TBSS.	Cross-sectional study; FA was measured in adults with ASD and TD to assess abnormalities in WM tracts. The MATRICS battery was used to explore potential relationships between measures of FA with a wide range of neurocognitive abilities. Assessment through ADOS, ADI-R, FSIQ ≥ 80.	Adults with ASD had ↓ FA in the ATR and the cingulum and exhibited poorer performance on many cognitive measures: processing speed, attention vigilance, working memory, visual learning, verbal learning, and social cognition. However, there was no significant relationship between FA in the ATR or the cingulum and any of the neuropsychological measures in either TD or ASD participants.	Abnormal cognitive function in ASD may be the effect of a different underlying mechanism from weaker diffusion in ATR and cingulum tracts (although they are the most impaired in ASD).
Ohta et al., 2020 [83]	105 participants with ASD (92 ♂ and 13 ♀), 55 with ADHD (42 ♂ and 13 ♀), and 58 TD (49 ♂ and 9 ♀), matched for age and sex. Clinical diagnosis of ASD and ADHD was made based on DSM-IV-TR. Sensory symptoms were evaluated using the subscale of AASP (ASD: *n* = 62, ADHD: *n* = 44, TD: *n* = 38).	3T MRI, DTI, TBSS.	One of the aims of this cross-sectional study was to examine the effect of a diagnosis of ASD and ADHD on DTI parameters. Using dimensional analyses, similarities in the brain–sensory symptoms relationship across diagnostic groups were examined. Interaction analysis was used to examine distinctions in brain–sensory symptoms relationships between diagnostic groups.	Compared with the TD group, in the posterior part of the CC, FA values were ↓ and RD values were ↑ in participants with ASD and ADHD, while they were not significantly different from each other. The dimensional analysis showed an area in the isthmus of the CC where the three groups had comparable relationships between the DTI parameters and sensory issues. In contrast, the interaction analyses showed, in the midbody of the CC, that the DD groups had a negative association between FA and SS, while the TD group showed a positive correlation. However, in the right posterior CC, participants with ASD had a positive correlation between RD value and SS scores, while individuals with ADHD had a negative correlation.	WM changes and their relationships to sensory issues are largely shared between ASD and ADHD.
Bletsch et al., 2020 [84]	92 participants with ASD (53 ♂ and 39 ♀) and 92 age-, sex-, and IQ-matched TD controls (51 ♂ and 41 ♀) aged 18–52 yrs.	3T MRI, DTI.	The main objectives of this cross-sectional study were to examine neuroanatomical differences at and around the GWM boundary in ASD participants relative to TD controls based on measures of diffusion, and to determine if alterations are modulated by sex and dependent on the examined tissue class (i.e., GM or WM) and cortical depth. Furthermore, to establish how variability in measures of diffusion sampled at the GWM boundary relates to regional differences in GWC in ASD participants. Assessment through ICD-10 and ADI-R.	Participants with ASD relative to TD controls displayed significant ↓ in FA and ↑ MD, especially at the GWM boundary. GWC significantly ↓ in ASD individuals, without any significant group × sex interaction effects. However, FA was ↑ in TD ♂ relative to TD ♀, while in the ASD group, ♀ had ↑ FA values compared to ♂. MD was ↓ in TD ♂ relative to TD ♀, while in ASD, MD was equal or slightly ↑ in ♂ compared to ♀. ↑ ASD symptoms associated with a more distinctive “blurring” of the GWM boundary and ↓ FA and ↑ MD at the GWM boundary. ASD-related neuroanatomical variation in diffusion features sampled at the GWM boundary overlapped to a large degree (about 50%) with differences in GWC.	This study provides evidence of neuroanatomical variations at the GWM boundary, as estimated by diffusion metrics, in ASD participants compared to TD controls.
Arunachalam Chandran et al., 2021 [85]	91 participants consisting of 66 TD and 25 ASD (52 ♂, 39 ♀, age 18–60 yrs, $\bar{x}$ AQ: 36.32 ASD and 14.86 TD). ASD diagnosis based on DSM-IV-TR and assessed with ADOS module-4. AQ scores were also collected from all participants. A subset of 53 participants consisting of 28 TD and 25 ASD matched for age, gender, and IQ took part in the DTI study.	3T MRI, SBM, VBM, TBSS	In this cross-sectional study, SBM was used to measure cortical thickness, surface area, and gyrification in the cortical grey matter, as well as VBM to characterise both cortical and subcortical grey matter volume at a whole-brain level. WM microstructure differences were studied using DTI. The relationship between these brain-based metrics and self-reported autistic traits assessed through AQ was explored.	Positive association was found between MD and AQ in the SLF, ILF, IFOF, and CC (forceps major and splenium), and a negative association between FA and AQ in the SLF, ILF, IFOF, and corticospinal tract. However, none of these clusters survived after correcting for multiple comparisons using threshold-free cluster enhancement. Autistic traits were found to be significantly associated with ↑ cortical thickness in the LLG, RLOC, RPT, and with ↑ surface area in the RLOC. Significantly associated clusters for ↑ local gyrification index were observed in the RLG. Significant positive association was found between regional GMV and AQ scores in subcortical brain regions, including the LP and RP, and significant negative association in the ROC, which also extended to the ACG.	These observations are consistent with previous results reported in case-control studies of ASD and demonstrate the value of using a dimensional approach.
Yoshikawa et al., 2022 [86]	63 participants with ASD (48 ♂, 15 ♀, $\bar{x}$ age 27.3 ± 5.6 yrs) and 38 TD participants (27 ♂, 11 ♀, $\bar{x}$ age 27.8 ± 5.6 yrs), matched for age and IQ, were assessed. ASD diagnosis was based on DSM-5 and ADOS-2 criteria, with traits evaluated via AQ-J. TD controls were screened using AQ-J (<32) and the MINI to exclude psychiatric history.	DTI, 3T MRI, 32-channel head coil, echo-planar imaging seq; T1-weighted for anatomic localisation.	Cross-sectional DTI study on ACE-related WM disruption in ASD compared to TD participants using the CATS to assess ACE severity, the AQ-J for autistic traits, and the ADOS-2 for diagnostic confirmation.	ASD group had ↓ yrs of education and ↑ AQ-J/CATS scores. ASD showed ↓ FA and ↑ RD in left ATR than TD group. High-CATS-score ASD had ↑ RD in left ATR vs. low-CATS-scores ASD and TD. CATS total and neglect/emotional abuse subscales correlated with ↓ FA and ↑ RD in left ATR/UF in ASD, while no correlations in TD group.	WM microstructural disruptions in left ATR were significant in ASD compared to TD, exacerbated by severe ACE exposure (neglect/emotional abuse). ASD individuals showed vulnerability to ACE-related effects on frontal-lobe tracts (ATR/UF), linked to cognitive dysfunction, emotional dysregulation, and psychiatric disorders vulnerability, with laterality favouring left-side disruption.
Cai et al., 2022 [87]	67 r-h adults: 32 ASD (20 ♂, 12 ♀; $\bar{x}$ age 27.98 ± 5.52 yrs, IQ ≥ 80) diagnosed via DSM-5 and ADOS-G; 35 TD (21 ♂, 14 ♀; $\bar{x}$ age 28.18 ± 5.53), matched for age, sex, and education, with no psychiatric/neurological history.	3T MRI, T1-weighted and DTI sequences processed with PANDA and FSL; FA maps generated using DTIFIT.	Cross-sectional study comparing WM structural networks in adults with ASD and TD controls using DTI and graph theory. Participants underwent clinical assessments (SRS, SCQ) and MRI scans processed to construct 90 × 90 FA-weighted WM networks using the AAL template. Global and regional metrics were analysed with GRETNA, and correlations with ASD symptoms were assessed.	No significant differences in age, sex, education, or IQ between the ASD and TD groups, except for lower operational IQ in the ASD group. ASD individuals showed reduced global efficiency and clustering coefficient but increased characteristic path length. Regionally, the ASD group had reduced nodal efficiency in the left precentral gyrus, left inferior frontal gyrus (triangle part), right precuneus, and right paracentral lobule. They showed increased nodal degree in the left frontal gyrus (opercular part), right supplementary motor area, and right postcentral gyrus. Altered network properties correlated with social responsiveness scores in the ASD group.	The study highlights topographical alterations in the WM structural network of adults with ASD, particularly in the frontal and parietal regions, reflecting impaired segregation and integration of brain network functions. These topological changes were found to correlate with the severity of clinical symptoms.
DiPiero et al., 2023 [88]	Total of 159 ♂ participants: 78 ASD ($\bar{x}$ age 26.66 ± 7.28 yrs) and 81 NT ($\bar{x}$ age 27.04 ± 6.83 yrs). ASD diagnosis was based on ADI-R, ADOS, and ICD-10 criteria; in addition, ADOS-2 was administered in the ASD group at timepoint 5. IQ was tested for all participants through WAIS-III.	3T MRI. T1-weighted MP2RAGE sequence; DTI and NODDI metrics derived via DIPY and DMIPY. TBSS and GBSS to analyse WM and GM microstructure voxelwise.	Cross-sectional study comparing WM and GM microstructure using TBSS and GBSS, respectively, between ASD and TD during late adolescence and adulthood. Age-related trajectories of diffusion metrics (derived from DTI and NODDI) were modelled using both linear and logarithmic functions, with the best-fitting model (based on AIC/BIC criteria) selected for further analysis. Group differences and age × group interactions were evaluated using GLMs, while non-parametric permutation testing (with TFCE correction) ensured robust statistical inference. Additionally, within the ASD cohort, microstructural metrics were correlated with clinical measures such as ADOS-2 CSS and SRS.	Age-related patterns of TBSS/GBSS dMRI metrics show FICVF and ODI ↑ with age, while MD, RD, and AD ↓; FA exhibits a flat trend. Across the TBSS and GBSS skeleton, age-related patterns are generally consistent with the global mean age-related trajectories for each measure, with slight differences for FA. Relationships with age in TBSS noted in tracts such as the fornix, anterior and posterior limbs of the internal capsules, external capsules, genu, body, and splenium of the CC, and anterior corona radiata, while in GBSS, skeleton relationships are consistently observed in insular and central opercular cortices and precentral and postcentral gyri. Among WM differences, the ASD group demonstrated ↓ FICVF and AD and ↑ ODI, MD, and RD compared to NT. FICVF, ODI, MD, and RD present differences between the groups in the anterior corona radiata and much of the CC. Regarding GM, the ASD group demonstrated ↑ ODI and ↓ FA compared to the NT group. ODI and FA are both noticed to differ between the groups in the right frontal pole, frontal orbital and insular cortices, and lingual and parahippocampal gyri. dMRI measures of ODI and FA significantly differed between groups in both WM and GM. WM and GM regions displaying significant group differences in ODI and FA include bilateral external capsules and insular cortices, bilateral posterior thalamic radiations and lingual gyri, the genu of the CC and cingulate gyri, left hemisphere cingulum bundle, and parahippocampal gyrus. Within the ASD cohort, TBSS reveals that ADOS-CSS positively correlated with WM for FA and AD in the genu and body of the CC (*p* < 0.05, FWER-corrected), after adjusting for age and IQ.	The study uses NODDI with TBSS and GBSS, observing significant WM and GM microstructural differences between ASD and NT from adolescence to adulthood, with altered FICVF, increased ODI, and decreased FA in key brain regions. WM microstructure correlates with autism severity (ADOS-CSS). No significant age-by-group interactions suggest that differences may be established before adolescence. Study limitations include the cross-sectional design and all-male sample.
Weerasekera et al., 2024 [89]	From ABIDE II and COBRE database: 28 r-h participants with ASD (28 ♂, $\bar{x}$ age 38.1 ± 16 yrs), 38 with SZ (28 ♂, 10 ♀, $\bar{x}$ age 37.9 ± 13 yrs), and matched NT (ASD-NT: 29 ♂, $\bar{x}$ age 39.6 ± 15 yrs; SZ-NT: 31 ♂, 10 ♀, $\bar{x}$ age 38.0 ± 12 yrs), matched for age, sex, and IQ. ASD diagnosis was based on ADOS-2 criteria, while SZ diagnosis followed SCID-IV criteria.	DTI, 3T MRI; T1-weighted for anatomic localisation.	A cross-sectional study using DTI and probabilistic tractography to study WM connectivity in adults with ASD and SZ compared to age- and IQ-matched NT controls. Data from the ABIDE II and COBRE databases were analysed to assess diffusivity parameters in the cingulate, orbitofrontal, and subcortical regions, focusing on differences related to social-cognitive and executive functions. SRS-2 and MSCEIT assessments were used as neuropsychological measures for ASD and SZ participants, respectively.	Distinct WM connectivity differences found in adults with ASD and SZ compared to NT controls. In ASD, ↑ MD and RD were observed between the left hippocampus and isthmus cingulate, along with longer path lengths in related tracts. The study also found ↓ AD and RD in specific cingulate–parahippocampal connections. In SZ, there were significant ↓ in FA in medial orbitofrontal–subcortical tracts and ↑ RD in putamen–medial orbitofrontal connections. Both disorders showed significant correlations between diffusion parameters and behavioural measures.	Distinct and overlapping WM connectivity differences in adults with ASD and SZ compared to NT. In ASD, ↑ MD and RD in the left Hip-IC and longer path lengths in related tracts were associated with full-IQ and SRS scores. In SZ, ↓ FA in medial orbitofrontal connections and ↑ RD in putamen–medial orbitofrontal tracts were linked to executive function and emotional regulation. Both disorders exhibited significant diffusivity differences predominantly in the right hemisphere but not the left isthmus cingulate, supporting the “dysconnectivity” hypothesis for social, emotional, and cognitive impairments. Limitations: small sample size, potential bias due to scanner-related differences across sites, and unequal sex distribution.
Shin et al., 2024 [90]	43 ASD (25 ♂, 18 ♀, $\bar{x}$ age 47.21 ± 10.86 yrs, range 30–73) and 43 NT (23 ♂, 20 ♀, $\bar{x}$ age 49.79 ± 12.01 yrs, range 30–70). ASD diagnosis was confirmed through AQ, SRS-2, ADOS-2, and expert clinical opinion following DSM-5 criteria. All participants were matched for age, sex, and IQ (assessed using WASI-II) and completed the RBS-R.	MRI, 3T, 64-channel head coil, using an echo-planar imaging sequence. Data were processed using FSL 6.0. A diffusion tensor model was applied to obtain FA and fwcFA maps. The study employed the TCATT for WM analysis and the MCALT for GM regions.	Cross-sectional study focusing on WM and GM microstructure using dMRI. A bi-tensor model was applied to quantify FW and fwcFA across 32 transcallosal WM tracts and 94 GM ROIs. Traditional single-tensor modelling was also used to estimate uncorrected FA in the same ROIs. The study hypothesized negligible FA and fwcFA differences but ↑ FW in WM for the ASD group relative to NT. It also examined age effects on dMRI metrics and assessed correlations between clinical measures (e.g., AQ, SRS-2, ADOS-2) and brain microstructure, predicting more pronounced age-related FW ↑ in ASD participants.	Among WM differences, ASD adults showed ↓ FA in 16 transcallosal tracts compared to NT, with PMv and preSMA surviving FDR correction. ↑ FW was observed in 24 tracts, frontal to occipital, all significant post-FDR. ↓ fwcFA in 6 occipital tracts, but none were significant. Among GM ROIs, ASD adults had ↓ FA in 31 ROIs, with only bilateral MCC and left SFG_med significant post-FDR. ↑ FW in 15 ROIs, with only the left MCC surviving FDR. ↓ fwcFA in 9 ROIs, but no significant differences. NT showed ↓ age-related FA across all WM tracts, while ASD adults showed this only in IFG_oper. ↑ Age-related FW were present in NT but absent in ASD. Post-FW correction, ↓ fwcFA remained in 11 frontal tracts in NT but not in ASD. Similar age-related FA and FW patterns were seen in GM ROIs, with no significant effects in ASD. ↑ FW in 24 WM tracts did not correlate with SRS-2, RBS-R, or ADOS-2. Higher AQ scores were linked to ↑ FW in IFG_orb and lateral orbital gyrus.	Globally elevated FW in 24 transcallosal WM tracts in ASD adults, while GM variations were negligible, except for the left MCC showing increased FW. Unlike NT adults, who showed age-related reductions in FA and increases in FW in both WM and GM, ASD adults showed negligible age-related changes, suggesting a heterogeneous brain aging profile. The study highlighted the importance of FW as a dMRI metric, as it provided insights into possible neuroinflammation, axonal degeneration, or altered immune responses in ASD adults. Correlations between elevated FW in the IFG_orb and lateral orbital gyrus and higher AQ scores suggest a link between FW and social-emotional regulation in ASD. Limitations: inclusion of only cognitively capable ASD adults, small sample size, and cross-sectional design.

* Incorporates data published two years later on the sample, in Roine et al., 2015 [91]. Abbreviations: AAA, adult assessment Asperger; AASP, Adolescent/Adult Sensory Profile; ACC, anterior cingulate cortex; ACE, adverse childhood experience; AD, axial diffusivity; ADI-R, Autism Diagnostic Interview–Revised; ADOS-2, Autism Diagnostic Observation Schedule second edition; ADOS-CSS, Autism Diagnostic Observation Schedule–Calibrated Severity Score; ADOS-G, Autism Diagnostic Observation Schedule–Generic; AK, axial kurtosis; ALIC, anterior limb of the internal capsule; ANOVA, analysis of variance; AQ, Autism Spectrum Quotient for Adults; AQ-J, Autism Spectrum Quotient Japanese version; ASD, autism spectrum disorder; ATR, anterior thalamic radiation; AxD, axial diffusivity; BDI, Beck’s Depression Inventory; BDI-II, Beck’s Depression Inventory-II; BVAQ, Bermond Vorst Alexithymia Questionnaire; CATS, Child Abuse and Trauma Scale; CC, corpus callosum; CG, central gyrus; CP, planarity coefficient; CSD, constrained spherical deconvolution-based tractography; DAN, dorsal attention network; DD, developmental disorder; dACC, dorsal anterior cingulate cortex; DIPY, diffusion imaging in Python; DKI, Diffusion Kurtosis Imaging; DMIPY, diffusion microstructure imaging in Python; DSM, Diagnostic and Statistical Manual of Mental Disorders; DTI, diffusion tensor imaging; DWI, diffusion-weighted imaging; EHI, Edinburgh Handedness Inventory; EPI, echo-planar imaging; EQ, Empathising Quotient; Eyes Test, Reading the Mind in the Eyes Test; FA, fractional anisotropy; FDR, false discovery rate; FICVF, intracellular volume fraction of neurites; FMa, forceps major; FMi, forceps minor; FOV, field of view; FPI, Freiburg Personality Inventory test; FRT, Benton Facial Recognition Test; FSIQ, Full-Scale IQ; FSL, FMRIB’s Software Library; FW, Free water; fwcFA, Free water-corrected FA; FWER, family-wise error rate; GBSS, grey matter-based spatial statistics; GLM, general linear model; GM, grey matter; GMV, grey matter volume; GWC, grey–white matter tissue contrast; GWM, grey–white matter boundary; ^1^H-MRS, Proton Magnetic Resonance Spectroscopy; Hip-IC, hippocampus–isthmus cingulate; ICC, intraclass correlation coefficient; ICD-10, International Classification of Diseases, 10th Edition; IFG_oper, inferior frontal gyrus-pars opercularis; IFG_orb, inferior frontal gyrus-pars orbitalis; IFOF, inferior fronto-occipital fasciculus; ILF, inferior longitudinal fasciculus; IQ, intelligence quotient; JART, Japanese version of the National Adult Reading Test; L-ALIC, left anterior limb of the internal capsule; LFPN, left-lateralised fronto-parietal network; lGI, local gyrification index; LICA, linked independent component analysis; LLG, left lingual gyrus; LP, left putamen; L-SLF, left superior longitudinal fasciculus; LST, left stria terminalis; MA, maternal age; MCALT, Mayo Clinic Adult Lifespan Template; MCC, middle cingulate cortex; MCP, middle cerebellar peduncle; MD, mean diffusivity; MFG, medial frontal lobe; MINI, Mini-International Neuropsychiatric Interview; MK, mean kurtosis; MNI, Montreal Neurological Institute; Mori WM atlas, Mori white-matter atlas; MPRAGE, Magnetization Prepared RApid Gradient Echo; MRI, magnetic resonance imaging; MSCEIT, Mayer–Salovey–Caruso Emotional Intelligence Test; NAA/Cr, N-acetylaspartate/creatine; NODDI, Neurite Orientation Dispersion and Density Imaging; NT, neurotypical; ODI, orientation dispersion index; PA, parental age; PANDA, Pipeline for Analyzing braiN Diffusion imAges; PCT, pontine crossing tract; Philips Ingenia, specific MRI scanner model; PIQ, performance IQ; preSMA, pre-supplementary motor area; PRESS, point-resolved spectroscopy sequence; Prtc(s), participant(s); PTR, posterior thalamic radiation; QEAS, empathy and appropriate social behaviour test; RAADS-R, Ritvo Autism Asperger Diagnostic Scale–Revised; RALIC, right anterior limb of the internal capsule; RBS-R, Repetitive Behaviour Scale-Revised; RD, radial diffusivity; R-ILF, right inferior longitudinal fasciculus; RK, radial kurtosis; RLG, right lingual gyrus; RLOC, right lateral occipital cortex; ROC, right orbitofrontal cortex; ROI, region of interest; RP, right putamen; RPT, right pars triangularis; RUF, right uncinate fasciculus; sACC, subgenual anterior cingulate cortex; SBM, surface-based morphometry; SCID-IV, Structured Clinical Interview for DSM-IV; SFG_med, superior frontal gyrus-medial; SLF, superior longitudinal fasciculus; SMA, supplementary motor area; SPM, statistical parametric mapping; SQ, systemising quotient; SRS, Social Responsiveness Scale; SS, sensory sensitivity; SW, software version; SZ, schizophrenia; T, Tesla; TBSS, tract-based spatial statistics; TCATT, transcallosal tractography template; TD, typically developed; TPJ, temporo–parietal junction; UF, uncinate fasciculus; VBM, voxel-based morphometry; VIQ, verbal IQ; vPMC, ventral premotor cortex; WAIS, Wechsler Adult Intelligence Scale; WAIS-III, Wechsler Adult Intelligence Scale–Third Edition; WAIS-R, Wechsler Adult Intelligence Scale–Revised; WASI, Wechsler Abbreviated Scale of Intelligence; WM, white matter; $\bar{x}$, mean; yr(s), year(s); ×, for, per; ±SD, standard deviation; ↓, decrease, decreased, lower, diminution, worsening; ↑, increase, augmentation, elevation, improvement, greater; →, followed by, subsequently, then; ♀, female, woman; ♂, male, man.

## 3. Results

The above search performed on 21 May 2025 produced 241 records on PubMed, 6 on PsycINFO, and 1 on CINAHL. Of these, a total of 26 studies, all identified by PubMed, met eligibility criteria and were included. Relevant data regarding study design, DTI metrics (e.g., FA, RD, MD, AK, etc.), and reported alterations (or no change) in individuals with ASD were extracted. No meta-analysis or individual-level data analysis was performed, as the focus of this review was on aggregate qualitative findings from eligible primary studies. Although the studies were remarkably homogeneous and no need to address heterogeneity was present, they differed in the reported outcomes.

**Figure 1:**
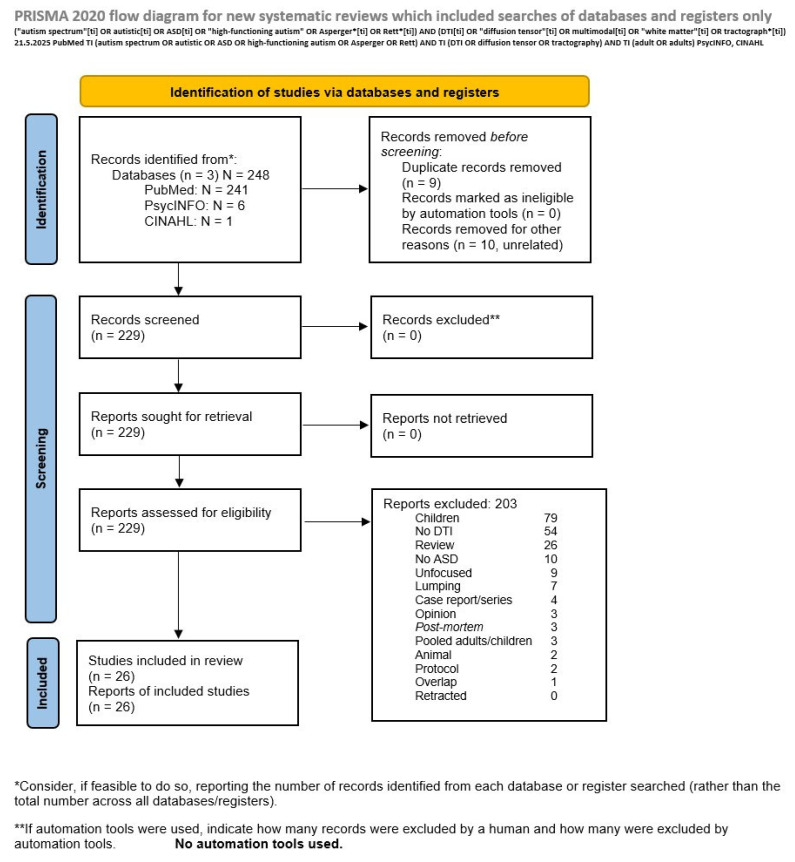
PRISMA 2020 flowchart of our search strategy. Source: Page MJ, et al. *BMJ* 2021; 372: n 71. https://doi.org/10.1136/bmj.n71 [64]. This work is licensed under CC BY 4.0. To view a copy of this license, visit https://creativecommons.org/licenses/by/4.0/, accessed on 28 July 2025.

All eligible studies were analysed, and their summary is provided in Table 1. The PRISMA 2020 flow diagram illustrates the selection process and provides details on the reasons for exclusion (Figure 1).

The first hypothesis of an exclusive WM alteration in ASD was published in May 1999, and the last published result was on 19 May 2025 (Supplementary Table S1). Eligible studies spanned from October 2010 [67] to 6 March 2025 [90]. Eligible studies are shown in Table 1 [59,60,67,68,69,70,71,72,73,74,75,76,77,78,79,80,81,82,83,84,85,86,87,88,89,90]. As for the research sites involved in these studies, most were in the US (N = 11), while Japan contributed with six and the UK with four. Three were conducted in the current EU—two in Germany and one in Finland—and one each in China and Australia [59,60,67,68,69,70,71,72,73,74,75,76,77,78,79,80,81,82,83,84,85,86,87,88,89,90] (Table 2). In one study, the authors were based in Iran, but they used four datasets from a US-based open-access database [81]. Most were carried out in single research sites, but two English studies involved a London and a Cambridge, UK site [75,84]. The last included study [90] involved an international collaborative database, which probably indicates strategies to adopt for the future. Of the six articles identified by PsycINFO, one was a Ph.D. thesis and five were PubMed duplicates (see Supplementary Table S1), and the one identified by CINAHL was a duplicate of both PubMed and PsycINFO.

The included studies involved 965 individuals with ASD, but of the studies, only one involved more than 100 individuals [83]. Of the involved individuals, 744 were male, constituting the majority of the sample (77.1%). The percentages loosely respect the 4:1 male-to-female distribution of ASD.

The machinery involved in image acquisition was, in all but two cases, 3 Tesla instruments. Both these exceptions used 1.5 Tesla instruments: one was the earliest study included, conducted by a mixed Dutch–British group located in London and published in 2010 [67]; the other was published in 2015 and conducted at Karasuyama Hospital’s outpatient services in Tokyo, Japan [59].

The detailed findings of the eligible studies, other than what is shown in Table 1 and Table 2, will be revealed in the Discussion section, where they will be interpreted in the light of other evidence.

### 3.1. Main Findings from Included Studies

We found extensive alterations in DTI measures in several WM pathways and reveal them below according to location.

### 3.2. Frontal and Interhemispheric Regions (Corpus Callosum [CC] and Frontal Tracts)

The most consistently reported alterations were found in commissural fibres, particularly in the CC. Multiple studies reported decreased FA and increased MD and RD in various CC segments, specifically in the genu, body, and splenium, suggesting reduced interhemispheric communication and myelin integrity in ASD [67,68,69,70,71,72,77,79,83,88,91]. Forceps minor, a frontal extension of the corpus callosum, anteriorly to the genu, also showed increased RD and MD [70,80]. Additionally, Shin et al. [90] observed elevated free water in seven frontal transcallosal tracts, i.e., in the gyrus rectus, medial orbital gyrus, olfactory cortex, dorsal and ventral premotor cortices, pre-supplementary motor area, and supplementary motor area; this may explain the functional deficits observed in the frontal lobe, to which the CC is connected. Higher FA and AD in the CC were positively associated with autism symptom severity [88]. WM damage in frontal tracts and the CC could partly explain the impairments in social and behavioural functioning observed in individuals with ASD [92]. Additionally, people with callosal agenesis exhibited deficits in emotion processing and higher-order cognitive tasks like those seen in individuals with ASD [93,94].

### 3.3. Superior Longitudinal Fasciculus and Other Association Fibres

Alterations in association fibres have been consistently reported in ASD. The ASD high-functioning group showed a significant decrease in FA in the anterior left superior longitudinal fasciculus (LSLF) and a significant increase in RD in the LSLF; this may suggest alterations in connectivity with the frontal lobe [76]. Another measure of DTI, AK, is reduced in ASD. AK is negatively correlated with autistic traits (AQ) and systemising traits (SQ) in the uncinate fasciculus (UF), inferior fronto-occipital fasciculus (IFOF), inferior longitudinal fasciculus (ILF), and superior longitudinal fasciculus (SLF) [79]. Furthermore, always in the SLF, but also the IFL, IFOF, and major forceps (occipital) and splenium of the CC, a positive association between MD and AQ was observed, along with a negative association between FA and AQ in the SLF, IFL, IFOF, and the corticospinal tract [85]. Furthermore, Yassin et al. [80] observed increased MD and RD in several association tracts (including the IFOF, SLF, and UF) as well as in the forceps minor, indicating widespread disruption of association fibres. Peeva et al. [73] found that, although the mean FA along the tract between the left supplementary motor area (SMA) and the ventral premotor cortex (vPMC) did not differ between ASD and NT individuals, the directional coherence between voxels along the tract was lower in individuals with ASD. Individuals with ASD showed lower FA and higher RD [80,86] and MD [80] in the ATR (anterior thalamic radiation), when compared to TD participants. ACE (adverse childhood experience) severity correlated with WM alterations in the left UF and ATR, with neglect linked to both and emotional abuse specifically affecting the left ATR [86].

Alterations in brain pathways such as the UF, ILF, and IFOF have been consistently associated with deficits in emotional processing, social interactions, and visual processing in both preschoolers [95] and adults [72,91,96,97]. These disruptions may help explain the social and emotional difficulties observed in individuals with ASD. Similarly, deficits in communication and higher-order cognitive functions were found to be linked to abnormalities in the SLF in a mixed childhood/adult sample [98]; this tract plays a crucial role in integrating information across the different brain regions involved in these processes.

### 3.4. Projection and Subcortical White-Matter Tracts

Alterations in projection fibres and subcortical tracts were also documented. Mohajer et al. [81] found reduced FA and increased MD in regions such as the left anterior limb of the internal capsule (L-ALIC) and the external capsules in ASD individuals with depressive symptoms, with FA correlating with depression scores. Haigh et al. [82] reported decreased FA in the ATR and the right cingulum, which was linked to poorer cognitive performance. Yoshikawa et al. [86] similarly observed lower FA and higher RD in the left ATR, with these alterations correlating with the severity of ACEs, correlations which were evident in both the left UF and ATR. DiPiero et al. [88] extended these findings to additional subcortical structures, reporting reduced neurite density (FICVF) and AD, alongside increased ODI, MD, and RD in tracts such as the fornix, the anterior and posterior limbs of the internal capsule, the external capsules, and the ATR. Weerasekera et al. [89] identified increases in MD and RD in the region connecting the hippocampus to the isthmus of the cingulate, as well as a longer path connecting the left pallidum with the isthmus, suggesting impaired limbic connectivity, which correlated with cognitive and communication measures.

Although alterations in projection and subcortical WM tracts were often reported in diffusion studies of ASD, their functional implications remain poorly understood. However, some evidence suggests that abnormalities in subcortical sensory–emotional circuits may contribute to core autistic traits. For example, Huang et al. [99] described alterations in the superior colliculus–pulvinar–amygdala pathway, a subcortical route involved in rapid emotional processing, suggesting that atypical connectivity in these regions could affect the ability to preconsciously detect socially salient stimuli. Furthermore, WM tracts involved in the control of repetitive behaviour in the basal ganglia may be related to the repetitive behaviour often observed in ASD individuals [100,101], and this may underlie the co-morbidity between ASD and obsessive–compulsive disorder [102,103].

### 3.5. Network Topology and Localised Measures

Some studies have taken a broader network approach. Cai et al. [87] analysed the topological properties of the WM structural network in ASD. ASD individuals showed decreased small-worldness and increased global efficiency (Eglob) compared to TD controls. The former exhibited reduced nodal efficiency (Enodal), such as in the left inferior frontal gyrus (IFG). Altered Eglob and Enodal in the left IFG correlated with social responsiveness scores (SRS), linking connectivity changes to ASD symptoms. Higher path length values indicated less efficient whole-brain information transmission, suggesting network disorganisation. Increased Eglob in ASD adults, unlike findings in children, may reflect neurodevelopmental changes improving long-distance connectivity.

Bletsch et al. [84] also reported differences in diffusion metrics at the grey–white-matter boundary (GWM). Increased ASD symptoms are associated with a more distinctive “blurring” of the GWM boundary, as well as lower FA and higher MD at the GWM boundary, suggesting that changes in tissue contrast at this interface may serve as a potential neuroanatomical marker. Another study [104] introduced the boundary sharpness coefficient (BSC) concept as a measure of microstructural alterations at the cortical GWM boundary. ASD individuals had significantly higher BSC values in the bilateral superior temporal gyrus and left IFG, indicating a more abrupt transition between white and grey matter intensities. An MRI study [105] found widespread cortical contrast reductions in ASD, correlating with ADOS scores. A Bayesian model predicted ASD with up to 86% accuracy using motor regions. These findings suggest that cortical contrast alterations are closely linked to ASD severity and can aid diagnostic prediction. In addition, adults with ASD showed increased local gyrification indices in the left pre- and post-central gyri corresponding to increased axial diffusivity of short WM tracts connecting these gyri [75].

### 3.6. Other Specific and Localised Findings

Additional studies have highlighted more localised white-matter alterations. Yamagata et al. [78] found no significant differences in standard DTI parameters between ASD individuals and unaffected siblings, except for an increase in ASD in the left stria terminalis, indicating that some abnormalities may be highly localised. Meanwhile, Kirkovski et al. [74] reported no significant group differences in DTI metrics, underscoring the heterogeneity of findings across studies.

These findings may reflect the marked heterogeneity of WM alterations observed across studies of ASD. As highlighted by Yeh et al. [106], such variability may be partly explained by methodological differences (e.g., tensor-based metrics being sensitive to crossing fibres), heterogeneous sampling (e.g., sex, head motion, and similar artefacts), and the overrepresentation of intellectually able individuals without psychiatric comorbidities, who represent only a subset of the autistic spectrum.

Summary of the most frequently reported white-matter alterations across the included DTI studies in autistic adults. Reductions in fractional anisotropy (FA) and increases in radial diffusivity (RD) are shown by tract. The number of studies reporting each finding is indicated in each column. Colour intensity reflects the consistency of reported alterations across studies.

**Table 2:** Study locations and sites involved in eligible studies.

Study	Location(s)	Site Number
Bloemen et al., 2010 [67]	King’s College London, UK	1
Thomas et al., 2011 [68]	Brain Imaging Research Center, University of Pittsburgh and Carnegie Mellon University, Pennsylvania, USA	1
Bakhtiari et al., 2012 [69]	Lausanne University Hospital, Lausanne, CH	1
Kleinhans et al., 2012 [70]	Department of Radiology, University of Washington, Seattle, Washington, USA	1
Mueller et al., 2013 [71]	Ludwig–Maximilians Universität München, Bayern, Deutschland, EU	1
Roine et al., 2013 [72]	Helsinki (NeuroMental) and Helsinki University Central Hospital neuropsychiatric clinics, Helsinki, Finland, EU	1
Peeva et al., 2013 [73]	Athinoula A. Martinos Center for Biomedical Imaging, MGH, Charlestown, Massachusetts, USA	1
Itahashi et al., 2015 [59]	Karasuyama Hospital, Tokyo, Japan	1
Libero et al., 2015 [60]	Civitan International Research Center, University of Alabama at Birmingham, Alabama, USA	1
Kirkovski et al., 2015 [74]	Brain and Psychological Science Research Centre, Swinburne University, Hawthorn, Victoria, Australia	1
Ecker et al., 2016 [75]	IoPPN, King’s College London, and Autism Research Centre, University of Cambridge, UK	2
Libero et al., 2016 [76]	University of Alabama at Birmingham (UAB), Civitan Sparks Autism Spectrum Disorders Clinic, UAB Medical Autism Clinic, University of Alabama Autism Spectrum Disorders Clinic, local community-based autism clinics, and greater Birmingham Community; presumably all MRIs conducted at Civitan	1
Nickel et al., 2017 [77]	Universitätsklinikum Freiburg, Freiburg im Bresgau, Baden–Württemberg, Deutschland, EU	1
Yamagata et al., 2018 [78]	Showa University, Tokyo, Japan	1
Hattori et al., 2019 [79]	Department of Radiology, Juntendo University Graduate School of Medicine and The University of Tokyo Graduate School of Medicine, Hongo, Bunkyo-ku, Tokyo, Japan	1
Yassin et al., 2019 [80]	The University of Tokyo Graduate School of Medicine, Hongo, Bunkyo-ku, Tokyo, Japan	1
Mohajer et al., 2019 [81]	Barrow Neurological Institute, Phoenix, Arizona, USA, four datasets from Autism Brain Imaging Data Exchange (ABIDE) II-Tehran and Zanjan, Iran	4
Haigh et al., 2020 [82]	Center for Excellence in Autism Research at the University of Pittsburgh, Pennsylvania, USA	1
Ohta et al., 2020 [83]	Medical Institute of Developmental Disabilities Research at Showa University, Tokyo, Japan	1
Bletsch et al., 2020 [84]	IoPPN, King’s College London, and Autism Research Centre, University of Cambridge, UK	2
Arunachalam Chandran et al., 2021 [85]	University of Reading, England, UK	1
Yoshikawa et al., 2022 [86]	Department of Psychiatry, Nara Medical University Hospital, Nara, Nara Prefecture, Japan	1
Cai et al., 2022 [87]	Department of Developmental Neuropsychology, School of Psychology, Army Medical University, Chongqing, China	1
DiPiero et al., 2023 [88]	University of Utah, Salt Lake City, Utah, USA	1
Weerasekera et al., 2024 [89]	BNI (Business Network International) and COBRE (Center for Biomedical Research and Education) at The Mind Research Network (MRN), Charlotte, North Carolina, USA	1
Shin et al., 2024 [90]	Center for Autism and Related Disabilities (CARD) at the University of Florida in Gainesville, University of Central Florida, University of South Florida, and the SPARK Research Match	1

## 4. Discussion

This review examined the literature on the integrity of WM fibre tracts in ASD adults using DTI. The objective was to investigate whether adults diagnosed with ASD exhibit alterations in WM measures, thus connectivity, compared to NT individuals, given that the structural connectivity between brain regions is increasingly recognised as a key factor in understanding the neurobiological mechanisms of ASD. Emphasis was placed on DTI as a non-invasive neuroimaging technique for assessing microstructural properties and the organisation of WM tracts.

In summary, the current literature demonstrates that WM alterations in ASD are regionally diverse, affecting frontal and interhemispheric tracts, association fibres, and subcortical projection pathways, and extend to network-level organisation. These alterations, reflected in parameters such as FA, MD, RD, AD, AK, FICVF, and ODI, may arise from disruptions in myelination, fibre coherence, and compensatory developmental mechanisms, thereby contributing to the cognitive, behavioural, and social deficits typically observed in autism.

**Figure 2:**
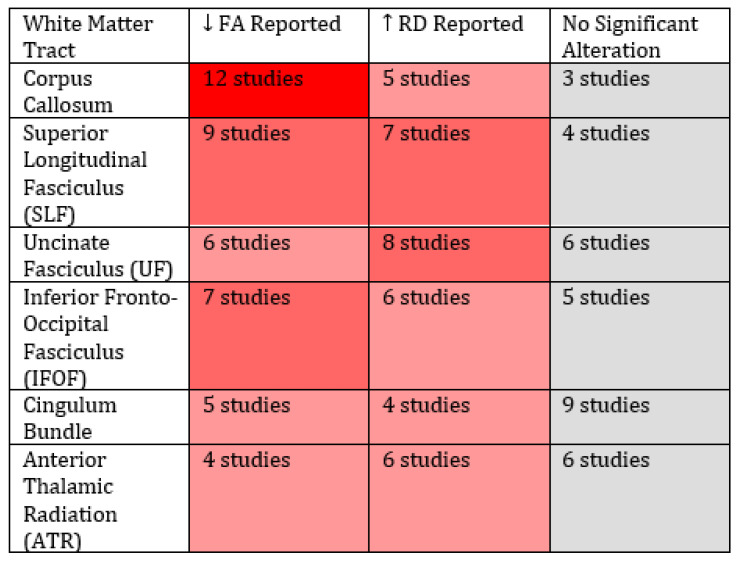
Summary of the most frequently reported white-matter alterations across the included DTI studies in autistic adults. Reductions in fractional anisotropy (FA) and increases in radial diffusivity (RD) are shown by tract. The number of studies reporting each finding is indicated in each column. Colour intensity reflects the consistency of reported alterations across studies. Alterations have been reported in several tracts, including the Corpus Callosum [50,59,67,71,74,75,76,77,80,83,84,88], Superior Longitudinal Fasciculus [69,70,72,76,78,85,87,88], Uncinate Fasciculus [67,69,71,72,78,83,86,88], Inferior Fronto-Occipital Fasciculus [67,69,71,72,80,85,88], Cingulum Bundle [70,76,82,84], and Anterior Thalamic Radiation [67,69,72,83,86,88].

To provide a clearer overview of the most consistently reported alterations, we created a summary figure (Figure 2) that synthesizes findings across the included studies. The figure highlights the frequency of significant alterations, such as reductions in fractional anisotropy (FA) and increases in radial diffusivity (RD), for key white-matter tracts, including the corpus callosum, superior longitudinal fasciculus, uncinate fasciculus, and inferior fronto-occipital fasciculus. The colour intensity in the figure reflects how frequently each tract was found to be altered across studies, facilitating the visual identification of the most affected fibre bundles in adults with ASD. A dedicated column also provides the specific references supporting the reported alterations, allowing for immediate traceability of the evidence base.

The overall quality of the studies we included may be deemed as satisfactory to good (Supplementary Table S2). While there are minor concerns in the first appearing studies with ethical approvals (often not reported or very generically referred to), the later studies fare better in this respect. Also, the locations where the experiments were performed were not precisely described in most studies.

To enhance the interpretability of the reported findings, we provide a conceptual framework summarizing the biological meaning and pathophysiological implications of the most commonly used DTI metrics. While many included studies reported alterations in FA, RD, MD, AD, and other advanced metrics such as axial kurtosis (AK), orientation dispersion index (ODI), and free water (FW), a unified interpretive approach is often lacking in the literature. As shown in Table 3, each DTI metric reflects specific microstructural properties of white matter. For example, FA represents the directionality of water diffusion and is sensitive to axonal organization and myelination. A reduction in FA often suggests axonal disorganization or demyelination. RD increases are typically associated with demyelination, whereas AD decreases may reflect axonal injury. MD, as an average of diffusion in all directions, is indicative of tissue integrity and can be elevated in the presence of inflammation, oedema, or loss of cellular density. More advanced metrics like AK and ODI (from neurite orientation dispersion and density imaging, or NODDI) provide further insights into microstructural complexity and neurite branching. FW, derived from bi-tensor models, serves as a marker of extracellular water content and may reflect neuroinflammatory processes. Together, these parameters offer a multidimensional view of white-matter integrity, allowing researchers to infer the potential mechanisms underlying the altered connectivity observed in autism ASD.

The observed attenuation or partial normalization of white-matter abnormalities in adults with ASD aligns with several theoretical models of atypical neurodevelopment. One such model is the delayed maturation hypothesis, which proposes that individuals with ASD follow typical neurodevelopmental trajectories, but at a slower pace. This may explain why some DTI abnormalities, such as reduced FA or elevated RD, are more pronounced in childhood and appear to diminish or stabilize in adulthood. Relatedly, the compensatory neurodevelopment model posits that alternative neural pathways or strategies are recruited over time to support cognitive and behavioural functioning, potentially mitigating the impact of early structural anomalies [25]. These compensatory mechanisms may involve increased engagement of prefrontal and associative networks, as supported by functional neuroimaging studies showing altered but task-effective brain activation in high-functioning adults with ASD.

Furthermore, the interactive specialization framework suggests that the functional specialization of neural systems emerges from dynamic interactions between brain regions during development, rather than through isolated maturation. Within this framework, atypical early connectivity patterns may gradually reorganize through experience-dependent plasticity, leading to more normalized DTI measures in adulthood. This could help explain why some adult individuals with ASD, particularly those with higher cognitive functioning, exhibit white-matter profiles closer to their neurotypical peers, despite persistent behavioural features. These models support the view that white-matter alterations in ASD are developmentally dynamic, and that observed adult patterns reflect a combination of early neurobiological vulnerability and adaptive developmental processes.

The synthesis of white-matter abnormalities in adult ASD has meaningful implications for clinical translation. From a diagnostic perspective, consistent alterations in DTI metrics, such as reduced fractional anisotropy in the corpus callosum or increased radial diffusivity in association fibres, may contribute to the identification of neurobiological signatures that distinguish ASD from other neurodevelopmental or psychiatric conditions. Although DTI is not yet used in routine clinical diagnostics, the convergence of findings across studies suggests that structural connectivity measures could be incorporated into multimodal diagnostic models, particularly when combined with behavioural or genetic data [22,31].

In terms of intervention, understanding the regional specificity of white-matter disruption may help guide targeted therapeutic approaches, such as cognitive remediation or neuromodulation techniques (e.g., transcranial magnetic stimulation) aimed at enhancing fronto-striatal or interhemispheric connectivity. Moreover, the dynamic nature of white-matter changes across the lifespan highlights a potential critical window for neuroplasticity-based interventions, especially in late adolescence and early adulthood, when certain DTI abnormalities appear to attenuate [35].

Finally, DTI-based metrics hold promise as candidate biomarkers for monitoring treatment response or stratifying subgroups within the autism spectrum. For instance, individuals with persistent callosal or limbic white-matter abnormalities may exhibit distinct clinical profiles or treatment trajectories. Future research should aim to validate these imaging markers longitudinally and explore their integration into precision psychiatry frameworks for ASD.

### Limitations

A key limitation of the reviewed literature lies in the methodological heterogeneity across studies, which complicates direct comparisons and the generalisation of findings. First, the magnetic field strength of MRI scanners varied across studies, with most using 3 Tesla instruments but some relying on older 1.5T systems, which offer lower signal-to-noise ratios and reduced spatial resolution. These differences can significantly impact the sensitivity of diffusion measurements, particularly in smaller or less myelinated white-matter tracts. Second, the DTI acquisition and processing techniques were highly variable. While some studies employed tract-based spatial statistics (TBSS) to enhance voxelwise alignment across subjects, others used region-of-interest (ROI) approaches, probabilistic tractography, or whole-brain voxel-based analyses. Each method carries specific strengths and limitations in terms of anatomical specificity, sensitivity to motion artefacts, and dependence on preprocessing parameters. Third, the diagnostic criteria and clinical assessment tools for ASD differed across studies, with some using DSM-IV, others DSM-5, and a range of instruments such as ADOS, ADI-R, or self-report scales. These discrepancies may lead to the inclusion of heterogeneous samples differing in symptom severity, comorbid conditions, or functional status, thus affecting the interpretation of neuroimaging findings.

Taken together, these sources of variability underscore the challenges of synthesising structural imaging studies in ASD and justify our choice of a systematic qualitative approach rather than a formal meta-analysis. Future research should aim for greater harmonisation in imaging protocols and diagnostic procedures to facilitate more robust cross-study comparisons and the identification of reproducible neurobiological markers.

Another limitation of our approach is that we did not relate the alterations found by the studies we included to other possible alterations, e.g., in grey matter, functional connectivity, and functional magnetic resonance imaging responses to specific tasks. The limitations of eligibility were duly acknowledged in each study and mainly regard the small sample sizes and the male-to-female discrepancy. The preponderance of male subjects in studies of autism is well known; this is reflected in the sex imbalance towards the male sex in such studies.

Some conclusions were contradictory; some studies showed intra-study incoherence besides inter-study differences. Notably, FA was reported as increased and decreased in the ASD sample in the same study [67]. While studies prior to 2013 pointed to decreased FA in ASD samples, Roine et al. [72,91] pointed out that there was actually an increase in FA in their study, contrasting previous results. These inconsistencies do not allow us to draw strong conclusions about the nature of WM alterations in adult ASD. The alterations in WM we encountered in the studies included in this review cannot be generalised to autistic individuals across the entire ASD spectrum.

While in some people, ASD may be detected early and show gross differences with non-ASD people regarding behaviour and relating to others, other people with ASD elude diagnosis until their adulthood. These people pose questions about having a disorder or just being differently developed and having their own, unique way of dealing with their environment. There are advocacy organisations that support the latter view or that avoid being involved in a potentially sterile and unfruitful debate [107], and others that stress the need for behavioural help and social inclusion [108]. Our study is unable to respond to the fundamental question of whether autism is a disorder or a different way of existing. Hypotheses on the reasons for neurodivergence have been advanced [109,110], but the question is far from being resolved. Current approaches do not dispute the inclusion of ASD among DSM-5-TR disorders, but as there is no large consensus on the use of medications [111,112] or psychotherapies for effectively treating ASD [113], psychotherapeutic approaches are currently proposed [114,115], while the main focus is on inclusion and societal changes in how people with ASD are viewed by TD individuals [18]. Regarding the differences between adult ASD and infantile/adolescent ASD, there is evidence of improvement from the paediatric age to adulthood. In fact, children with ASD show more pronounced and widespread WM disturbances, particularly in language-related tracts [116], while adults show less severe WM alterations, suggesting that the brain’s development still proceeds during adulthood, thus converging to typically developing patterns over time [117].

Future studies need to address the above discrepancies and employ multimodal approaches that need to be consistent to ensure study comparability. Although this review could not identify a precise WM signature of ASD, combining DTI with other techniques could disentangle the intricate processes that lead to altered function in people with autism.

## 5. Conclusions

The literature included in this review highlighted regionally diverse WM alterations in adult ASD, specifically in FA, MD, RD, AD, AK, FICVF, and ODI, compared to TD individuals, mostly in frontal and interhemispheric tracts, association fibres, and subcortical projection pathways, namely ATR, CC, IFOF, ILF, SLF, PTR, UF, and corticospinal tract. DTI parameter changes may reflect disrupted myelination and fibre coherence and may represent compensatory developmental mechanisms. The alterations found here are less pronounced than those found in children and adolescents, which is in line with studies comparing paediatric with adult autism, which indicate that some aspects of WM alterations in ASD improve during the brain maturation period. The persisting DTI alterations in ASD may contribute to the typical cognitive, behavioural, and social deficits of this disorder.

## Figures and Tables

**Table 3:** Conceptual framework linking DTI metrics to biological and pathophysiological interpretations.

DTI (Diffusion Tensor Imaging) Metric	Definition	Biological Interpretation	Possible Pathophysiological Mechanism
FA (Fractional Anisotropy)	Degree of directionality of water diffusion	Reflects axonal density, coherence, and myelination	↓ FA: axonal disorganization, demyelination
RD (Radial Diffusivity)	Diffusion perpendicular to axonal fibres	Sensitive to myelin integrity	↑ RD: demyelination or reduced myelin sheath
AD (Axial Diffusivity)	Diffusion parallel to axonal fibres	Related to axonal integrity	↓ AD: axonal damage or loss
MD (Mean Diffusivity)	Average diffusion in all directions	Reflects overall tissue density and membrane integrity	↑ MD: increased extracellular space, reduced cellularity
AK (Axial Kurtosis)	Non-Gaussianity of diffusion along axons	Sensitive to axonal complexity and restriction	↓ AK: reduced axonal microstructure complexity
ODI (Orientation Dispersion Index)	Degree of dispersion of fibre orientations (NODDI metric)	Reflects the branching and angular complexity of neurites	↑ ODI: greater orientation dispersion, possible immature connectivity
FW (Free Water)	Extracellular water fraction (bi-tensor model)	Marker of neuroinflammation or extracellular fluid increase	↑ FW: neuroinflammatory processes or oedema

Note: Increases (↑) or decreases (↓) in DTI metrics may not be specific and should be interpreted in the context of region, clinical profile, and complementary imaging findings.

## Data Availability

Not applicable. All data are in the manuscript/Supplemental Material and in cited articles.

## Supplementary Materials

The following supporting information can be downloaded at https://www.mdpi.com/article/10.3390/brainsci15080824/s1: Supplementary Table S1: PubMed search results with reasons for exclusion and eligibility (included). Supplementary Table S2: Quality Assessment for Diverse Studies (QuADS)—scale and scores attributed according to each criterion to each study. Supplementary Table S3: PRISMA 2020 checklist.

## Abbreviations

The following abbreviations are used in this manuscript:

AK	Axial Kurtosis
ASD	Autism Spectrum Disorder
ATR	Anterior Thalamic Radiation
CC	Corpus Callosum
DTI	Diffusion Tensor Imaging
FA	Fractional Anisotropy
FW	Free Water
GM	Grey Matter
GWM	Grey–White Matter Boundary
IFOF	Inferior Fronto-Occipital Fasciculus
ILF	Inferior Longitudinal Fasciculus
MD	Mean Diffusivity
MRI	Magnetic Resonance Imaging
NT	Neurotypical Individuals
ODI	Orientation Dispersion Index
PTR	Posterior Thalamic Radiation
RD	Radial Diffusivity
SLF	Superior Longitudinal Fasciculus
SMA	Supplementary Motor Area
SPM	Statistical Parametric Mapping
TBSS	Tract-Based Spatial Statistics
SZ	Schizophrenia
TD	Typically Developing Individuals
UF	Uncinate Fasciculus
VBM	Voxel-based Morphometry
WM	White Matter
