# Supplementary material for: Exploring the Autistic Brain: A Systematic Review of Diffusion Tensor Imaging Studies on Neural Connectivity in Autism Spectrum Disorder
Source: Brain Sci. 2025 Jul 31;15(8):824. doi: 10.3390/brainsci15080824 (PMC12384897; doi:10.3390/brainsci15080824)
Supplement: Supplementary file 1 [file brainsci-15-00824-s001.zip › brainsci-3729157-supplementary.pdf]

Supplementary Table S1. PubMed search results with reasons for exclusion and eligibility (included).

**PubMed**	("autism spectrum"[ti] OR autistic[ti] OR ASD[ti] OR "high-functioning autism" OR Asperger*[ti] OR Rett*[ti]) AND (DTI[ti] OR "diffusion tensor"[ti] OR multimodal[ti] OR "white matter"[ti] OR tractograph*[ti]) PubMed 21.5.2025 → 241 records	
1	Ellis HD, Gunter HL. Asperger syndrome: a simple matter of white matter? Trends Cogn Sci. 1999;3(5):192-200. doi: 10.1016/s1364-6613(99)01315-7.	Opinion
2	Herbert MR, Ziegler DA, Deutsch CK, O'Brien LM, Lange N, Bakardjiev A, Hodgson J, Adrien KT, Steele S, Makris N, Kennedy D, Harris GJ, Caviness VS Jr. Dissociations of cerebral cortex, subcortical and cerebral white matter volumes in autistic boys. Brain. 2003;126(Pt 5):1182-92. doi: 10.1093/brain/awg110.	No DTI
3	Herbert MR, Ziegler DA, Makris N, Filipek PA, Kemper TL, Normandin JJ, Sanders HA, Kennedy DN, Caviness VS Jr. Localization of white matter volume increase in autism and developmental language disorder. Ann Neurol. 2004 Apr;55(4):530-40. doi: 10.1002/ana.20032.	No DTI
4	Waiter GD, Williams JH, Murray AD, Gilchrist A, Perrett DI, Whiten A. Structural white matter deficits in high-functioning individuals with autistic spectrum disorder: a voxel-based investigation. Neuroimage. 2005;24(2):455-61. doi: 10.1016/j.neuroimage.2004.08.049.	No DTI
5	Alexander AL, Lee JE, Lazar M, Boudos R, DuBray MB, Oakes TR, Miller JN, Lu J, Jeong EK, McMahon WM, Bigler ED, Lainhart JE. Diffusion tensor imaging of the corpus callosum in Autism. Neuroimage. 2007;34(1):61-73. doi: 10.1016/j.neuroimage.2006.08.032. Epub 2006 Oct 4.	Children
6	Wong V. Occipital deep white matter hyperintensities in autism spectrum disorder. Pediatr Int. 2007;49(4):513-5. doi: 10.1111/j.1442-200X.2007.02411.x.	Case
7	Sundaram SK, Kumar A, Makki MI, Behen ME, Chugani HT, Chugani DC. Diffusion tensor imaging of frontal lobe in autism spectrum disorder. Cereb Cortex. 2008;18(11):2659-65. doi: 10.1093/cercor/bhn031. Epub 2008 Mar 20.	Children
8	Ke X, Tang T, Hong S, Hang Y, Zou B, Li H, Zhou Z, Ruan Z, Lu Z, Tao G, Liu Y. White matter impairments in autism, evidence from voxel-based morphometry and diffusion tensor imaging. Brain Res. 2009;1265:171-7. doi: 10.1016/j.brainres.2009.02.013. Epub 2009 Feb 21.	Children
9	McAlonan GM, Cheung C, Cheung V, Wong N, Suckling J, Chua SE. Differential effects on white-matter systems in high-functioning autism and Asperger's syndrome. Psychol Med. 2009;39(11):1885-93. doi: 10.1017/S0033291709005728. Epub 2009 Apr 9.	Children
10	Pugliese L, Catani M, Ameis S, Dell'Acqua F, Thiebaut de Schotten M, Murphy C, Robertson D, Deeley Q, Daly E, Murphy DG. The anatomy of extended limbic pathways in Asperger syndrome: a preliminary diffusion tensor imaging tractography study. Neuroimage. 2009;47(2):427-34. doi: 10.1016/j.neuroimage.2009.05.014. Epub 2009 May 14.	Pooled
11	Brito AR, Vasconcelos MM, Domingues RC, Hygino da Cruz LC Jr, Rodrigues Lde S, Gasparetto EL, Calçada CA. Diffusion tensor imaging findings in school-aged autistic children. J Neuroimaging. 2009;19(4):337-43. doi: 10.1111/j.1552-6569.2009.00366.x.	Children
12	Pardini M, Garaci FG, Bonzano L, Roccatagliata L, Palmieri MG, Pompili E, Coniglione F, Krueger F, Ludovici A, Floris R, Benassi F, Emberti Gialloreti L. White matter reduced streamline coherence in young men with autism and mental retardation. Eur J Neurol. 2009;16(11):1185-90. doi: 10.1111/j.1468-1331.2009.02699.x. Epub 2009 Jun 15.	Pooled
13	Mahmood A, Bibat G, Zhan AL, Izbudak I, Farage L, Horska A, Mori S, Naidu S. White matter impairment in Rett syndrome: diffusion tensor imaging study with clinical correlations. AJNR Am J Neuroradiol. 2010 Feb;31(2):295-9. doi: 10.3174/ajnr.A1792. Epub 2009 Oct 15.	Children
14	Knaus TA, Silver AM, Kennedy M, Lindgren KA, Dominick KC, Siegel J, Tager- Flusberg H. Language laterality in autism spectrum disorder and typical controls: a functional, volumetric, and diffusion tensor MRI study. Brain Lang. 2010;112(2):113-20. doi: 10.1016/j.bandl.2009.11.005. Epub 2009 Dec 23.	Children
15	Cheng Y, Chou KH, Chen IY, Fan YT, Decety J, Lin CP. Atypical development of white matter microstructure in adolescents with autism spectrum disorders. Neuroimage. 2010;50(3):873-82. doi: 10.1016/j.neuroimage.2010.01.011. Epub 2010 Jan 11.	Children
16	Sivaswamy L, Kumar A, Rajan D, Behen M, Muzik O, Chugani D, Chugani H. A diffusion tensor imaging study of the cerebellar pathways in children with autism spectrum disorder. J Child Neurol. 2010;25(10):1223-31. doi: 10.1177/0883073809358765. Epub 2010 Feb 22.	Children
17	Carmody DP, Lewis M. Regional white matter development in children with autism spectrum disorders. Dev Psychobiol. 2010;52(8):755-63. doi: 10.1002/dev.20471.	No DTI
**18**	**Bloemen OJ, Deeley Q, Sundram F, Daly EM, Barker GJ, Jones DK, van Amelsvoort TA, Schmitz N, Robertson D, Murphy KC, Murphy DG. White matter integrity in Asperger syndrome: a preliminary diffusion tensor magnetic resonance imaging study in adults. Autism Res. 2010;3(5):203-13. doi: 10.1002/aur.146. Erratum in: Autism Res. 2011;4(2):160.**	**Included**
19	Nizamie A, Sengupta U, Mishra BR, Praharaj SK, Nizamie SH. Role of early multimodal interventions in a case with autistic regression. Acta Neurol Taiwan. 2010;19(1):51-6.	Case
20	Avino TA, Hutsler JJ. Abnormal cell patterning at the cortical gray-white matter boundary in autism spectrum disorders. Brain Res. 2010;1360:138-46. doi: 10.1016/j.brainres.2010.08.091. Epub 2010 Sep 25.	*Post mortem*
**21**	**Thomas C, Humphreys K, Jung KJ, Minshew N, Behrmann M. The anatomy of the callosal and visual-association pathways in high-functioning autism: a DTI tractography study. Cortex. 2011;47(7):863-73. doi: 10.1016/j.cortex.2010.07.006. Epub 2010 Aug 3.**	**Included**
22	Noriuchi M, Kikuchi Y, Yoshiura T, Kira R, Shigeto H, Hara T, Tobimatsu S, Kamio Y. Altered white matter fractional anisotropy and social impairment in children with autism spectrum disorder. Brain Res. 2010;1362:141-9. doi: 10.1016/j.brainres.2010.09.051. Epub 2010 Sep 18.	Children
23	Jones CRG, Pickles A, Falcaro M, Marsden AJ, Happé F, Scott SK, Sauter D, Tregay J, Phillips RJ, Baird G, Simonoff E, Charman T. A multimodal approach to emotion recognition ability in autism spectrum disorders. J Child Psychol Psychiatry. 2011;52(3):275-85. doi: 10.1111/j.1469-7610.2010.02328.x. Epub 2010 Oct 18.	No DTI
24	Groen WB, Buitelaar JK, van der Gaag RJ, Zwiers MP. Pervasive microstructural abnormalities in autism: a DTI study. J Psychiatry Neurosci. 2011;36(1):32-40. doi: 10.1503/jpn.090100.	Children
25	Shukla DK, Keehn B, Müller RA. Tract-specific analyses of diffusion tensor imaging show widespread white matter compromise in autism spectrum disorder. J Child Psychol Psychiatry. 2011;52(3):286-95. doi: 10.1111/j.1469-7610.2010.02342.x. Epub 2010 Nov 12.	Children
26	Radua J, Via E, Catani M, Mataix-Cols D. Voxel-based meta-analysis of regional white-matter volume differences in autism spectrum disorder versus healthy controls. Psychol Med. 2011;41(7):1539-50. doi: 10.1017/S0033291710002187. Epub 2010 Nov 16.	Review
27	Shukla DK, Keehn B, Lincoln AJ, Müller RA. White matter compromise of callosal and subcortical fiber tracts in children with autism spectrum disorder: a diffusion tensor imaging study. J Am Acad Child Adolesc Psychiatry. 2010;49(12):1269-78, 1278.e1-2. doi: 10.1016/j.jaac.2010.08.018. Epub 2010 Oct 14.	Children
28	Jou RJ, Jackowski AP, Papademetris X, Rajeevan N, Staib LH, Volkmar FR. Diffusion tensor imaging in autism spectrum disorders: preliminary evidence of abnormal neural connectivity. Aust N Z J Psychiatry. 2011;45(2):153-62. doi: 10.3109/00048674.2010.534069. Epub 2010 Dec 6.	Children
29	Shukla DK, Keehn B, Smylie DM, Müller RA. Microstructural abnormalities of short-distance white matter tracts in autism spectrum disorder. Neuropsychologia. 2011;49(5):1378-1382. doi: 10.1016/j.neuropsychologia.2011.02.022. Epub 2011 Feb 17.	Children
30	Voineskos AN, Lett TA, Lerch JP, Tiwari AK, Ameis SH, Rajji TK, Müller DJ, Mulsant BH, Kennedy JL. Neurexin-1 and frontal lobe white matter: an overlapping intermediate phenotype for schizophrenia and autism spectrum disorders. PLoS One. 2011;6(6):e20982. doi: 10.1371/journal.pone.0020982. Epub 2011 Jun 8.	No ASD
31	Jeong J-W, Kumar AK, Sundaram SK, Chugani HT, Chugani DC. Sharp curvature of frontal lobe white matter pathways in children with autism spectrum disorders: tract-based morphometry analysis. AJNR Am J Neuroradiol. 2011;32(9):1600-6. doi: 10.3174/ajnr.A2557. Epub 2011 Jul 14.	Children
32	Cheon K-A, Kim Y-S, Oh S-H, Park S-Y, Yoon H-W, Herrington J, Nair A, Koh Y-J, Jang D-P, Kim Y-B, Leventhal BL, Cho Z-H, Castellanos FX, Schultz RT. Involvement of the anterior thalamic radiation in boys with high functioning autism spectrum disorders: a Diffusion Tensor Imaging study. Brain Res. 2011;1417:77-86. doi: 10.1016/j.brainres.2011.08.020. Epub 2011 Aug 16.	Children
33	Pina-Camacho L, Villero S, Fraguas D, Boada L, Janssen J, Navas-Sánchez FJ, Mayoral M, Llorente C, Arango C, Parellada M. Autism spectrum disorder: does neuroimaging support the DSM-5 proposal for a symptom dyad? A systematic review of functional magnetic resonance imaging and diffusion tensor imaging studies. J Autism Dev Disord. 2012;42(7):1326-41. doi: 10.1007/s10803-011-1360-4.	Review
34	Hong S, Ke X, Tang T, Hang Y, Chu K, Huang H, Ruan Z, Lu Z, Tao G, Liu Y. Detecting abnormalities of corpus callosum connectivity in autism using magnetic resonance imaging and diffusion tensor tractography. Psychiatry Res. 2011;194(3):333-339. doi: 10.1016/j.pscychresns.2011.03.009. Epub 2011 Nov 1.	Children
35	Bode MK, Mattila ML, Kiviniemi V, Rahko J, Moilanen I, Ebeling H, Tervonen O, Nikkinen J. White matter in autism spectrum disorders – evidence of impaired fiber formation. Acta Radiol. 2011;52(10):1169-74. doi: 10.1258/ar.2011.110197. Epub 2011 Nov 18.	Children
36	Ameis SH, Fan J, Rockel C, Voineskos AN, Lobaugh NJ, Soorya L, Wang AT, Hollander E, Anagnostou E. Impaired structural connectivity of socio-emotional circuits in autism spectrum disorders: a diffusion tensor imaging study. PLoS One. 2011;6(11):e28044. doi: 10.1371/journal.pone.0028044. Epub 2011 Nov 23.	Children
37	Duerden EG, Mak-Fan KM, Taylor MJ, Roberts SW. Regional differences in grey and white matter in children and adults with autism spectrum disorders: an activation likelihood estimate (ALE) meta-analysis. Autism Res. 2012;5(1):49-66. doi: 10.1002/aur.235. Epub 2011 Dec 2.	Review
**38**	**Bakhtiari R, Zürcher NR, Rogier O, Russo B, Hippolyte L, Granziera C, Araabi BN, Nili Ahmadabadi M, Hadjikhani N. Differences in white matter reflect atypical developmental trajectory in autism: A Tract-based Spatial Statistics study. Neuroimage Clin. 2012;1(1):48-56. doi: 10.1016/j.nicl.2012.09.001.**	**Included**
39	Iidaka T, Miyakoshi M, Harada T, Nakai T. White matter connectivity between superior temporal sulcus and amygdala is associated with autistic trait in healthy humans. Neurosci Lett. 2012;510(2):154-8. doi: 10.1016/j.neulet.2012.01.029. Epub 2012 Jan 25.	No ASD

40	Jeong JW, Chugani DC, Behen ME, Tiwari VN, Chugani HT. Altered white matter structure of the dentatorubrothalamic pathway in children with autistic spectrum disorders. Cerebellum. 2012;11(4):957-71. doi: 10.1007/s12311-012-0369-3.	Children
41	Mak-Fan KM, Morris D, Vidal J, Anagnostou E, Roberts W, Taylor MJ. White matter and development in children with an autism spectrum disorder. Autism. 2013;17(5):541-57. doi: 10.1177/1362361312442596. Epub 2012 Jun 14.	Children
42	White SW, Ollendick T, Albano AM, Oswald D, Johnson C, Southam-Gerow MA, Kim I, Scahill L. Randomized controlled trial: Multimodal Anxiety and Social Skill Intervention for adolescents with autism spectrum disorder. J Autism Dev Disord. 2013;43(2):382-94. doi: 10.1007/s10803-012-1577-x.	No DTI
43	Travers BG, Adluru N, Ennis C, Tromp do PM, Destiche D, Doran S, Bigler ED, Lange N, Lainhart JE, Alexander AL. Diffusion tensor imaging in autism spectrum disorder: a review. Autism Res. 2012;5(5):289-313. doi: 10.1002/aur.1243. Epub 2012 Jul 11.	Review
**44**	**Kleinhans NM, Pauley G, Richards T, Neuhaus E, Martin N, Corrigan NM, Shaw DW, Estes A, Dager SR. Age-related abnormalities in white matter microstructure in autism spectrum disorders. Brain Res. 2012;1479:1-16. doi: 10.1016/j.brainres.2012.07.056. Epub 2012 Aug 10.**	**Included**
45	Durand T, De Felice C, Signorini C, Oger C, Bultel-Poncé V, Guy A, Galano JM, Leoncini S, Ciccoli L, Pecorelli A, Valacchi G, Hayek J. F_2-Dihomo-isoprostanes and brain white matter damage in stage 1 Rett syndrome. Biochimie. 2013;95(1):86-90. doi: 10.1016/j.biochi.2012.09.017. Epub 2012 Sep 23.	No DTI
46	Billeci L, Calderoni S, Tosetti M, Catani M, Muratori F. White matter connectivity in children with autism spectrum disorders: a tract-based spatial statistics study. BMC Neurol. 2012;12:148. doi: 10.1186/1471-2377-12-148.	Children
47	Lerner MD, McPartland JC, Morris JP. Multimodal emotion processing in autism spectrum disorders: an event-related potential study. Dev Cogn Neurosci. 2013;3:11-21. doi: 10.1016/j.dcn.2012.08.005. Epub 2012 Sep 1.	No DTI
48	Vomstein K, Stieltjes B, Poustka L. Strukturelle Konnektivität und Diffusionstensor-Bildgebung bei Autismus-Spektrum-Störungen [Structural connectivity and diffusion tensor imaging in autism spectrum disorders]. Z Kinder Jugendpsychiatr Psychother. 2013;41(1):59-68. German. doi: 10.1024/1422-4917/a000210.	Review
49	Abdel Razek A, Mazroa J, Baz H. Assessment of white matter integrity of autistic preschool children with diffusion weighted MR imaging. Brain Dev. 2014;36(1):28-34. doi: 10.1016/j.braindev.2013.01.003. Epub 2013 Feb 8.	No DTI
50	Ellmore TM, Li H, Xue Z, Wong ST, Frye RE. Tract-based spatial statistics reveal altered relationship between non-verbal reasoning abilities and white matter integrity in autism spectrum disorder. J Int Neuropsychol Soc. 2013;19(6):723-8. doi: 10.1017/S1355617713000325. Epub 2013 Apr 8.	Children
51	Jakab A, Emri M, Spisak T, Szeman-Nagy A, Beres M, Kis SA, Molnar P, Berenyi E. Autistic traits in neurotypical adults: correlates of graph theoretical functional network topology and white matter anisotropy patterns. PLoS One. 2013;8(4):e60982. doi: 10.1371/journal.pone.0060982.	No ASD
52	Aoki Y, Abe O, Nippashi Y, Yamasue H. Comparison of white matter integrity between autism spectrum disorder subjects and typically developing individuals: a meta-analysis of diffusion tensor imaging tractography studies. Mol Autism. 2013;4(1):25. doi: 10.1186/2040-2392-4-25.	Review
**53**	**Mueller S, Keeser D, Samson AC, Kirsch V, Blautzik J, Grothe M, Erat O, Hegenloh M, Coates U, Reiser MF, Hennig-Fast K, Meindl T. Convergent Findings of Altered Functional and Structural Brain Connectivity in Individuals with High Functioning Autism: A Multimodal MRI Study. PLoS One. 2013;8(6):e67329. doi: 10.1371/journal.pone.0067329.**	**Included**
54	Aoki Y, Abe O, Nippashi Y, Yamasue H. Comparison of white matter integrity between autism spectrum disorder subjects and typically developing individuals: a meta-analysis of diffusion tensor imaging tractography studies. Mol Autism. 2013;4(1):25. doi: 10.1186/2040-2392-4-25.	Review
55	Joseph RM, Fricker Z, Fenoglio A, Lindgren KA, Knaus TA, Tager-Flusberg H. Structural asymmetries of language-related gray and white matter and their relationship to language function in young children with ASD. Brain Imaging Behav. 2014;8(1):60-72. doi: 10.1007/s11682-013-9245-0.	Children
56	Tamas D, Marković S, Milankov V. Systemic multimodal approach to speech therapy treatment in autistic children. Med Pregl. 2013;66(5-6):233-9. doi: 10.2298/mpns1306233t.	No DTI
57	Cauda F, Costa T, Palermo S, D'Agata F, Diano M, Bianco F, Duca S, Keller R. Concordance of white matter and gray matter abnormalities in autism spectrum disorders: a voxel-based meta-analysis study. Hum Brain Mapp. 2014;35(5):2073-98. doi: 10.1002/hbm.22313. Epub 2013 Jul 26.	Review
58	Roberts TP, Lanza MR, Dell J, Qasmieh S, Hines K, Blaskey L, Zarnow DM, Levy SE, Edgar JC, Berman JI. Maturational differences in thalamocortical white matter microstructure and auditory evoked response latencies in autism spectrum disorders. Brain Res. 2013;1537:79-85. doi: 10.1016/j.brainres.2013.09.011. Epub 2013 Sep 18.	Children
**59**	**Roine U, Roine T, Salmi J, Nieminen-Von Wendt T, Leppämäki S, Rintahaka P, Tani P, Leemans A, Sams M. Increased coherence of white matter fiber tract organization in adults with Asperger syndrome: a diffusion tensor imaging study. Autism Res. 2013;6(6):642-50. doi: 10.1002/aur.1332. Epub 2013 Oct 2.**	**Included**
60	Bergeron JD, Deslauriers J, Grignon S, Fortier LC, Lepage M, Stroh T, Poyart C, Sébire G. White matter injury and autistic-like behavior predominantly affecting male rat offspring exposed to group B streptococcal maternal inflammation. Dev Neurosci. 2013;35(6):504-15. doi: 10.1159/000355656. Epub 2013 Nov 13.	Animal
**61**	**Peeva MG, Tourville JA, Agam Y, Holland B, Manoach DS, Guenther FH. White matter impairment in the speech network of individuals with autism spectrum disorder. Neuroimage Clin. 2013;3:234-41. doi: 10.1016/j.nicl.2013.08.011.**	**Included**
62	Koldewyn K, Yendiki A, Weigelt S, Gweon H, Julian J, Richardson H, Malloy C, Saxe R, Fischl B, Kanwisher N. Differences in the right inferior longitudinal fasciculus but no general disruption of white matter tracts in children with autism spectrum disorder. Proc Natl Acad Sci U S A. 2014;111(5):1981-6. doi: 10.1073/pnas.1324037111. Epub 2014 Jan 21.	Children
63	Chase A. Brain imaging White matter disruption in autism spectrum disorder is exaggerated by head movements during neuroimaging. Nat Rev Neurol. 2014;10(3):122. doi: 10.1038/nrneurol.2014.20. Epub 2014 Feb 11.	Opinion
64	Jeong JW, Tiwari VN, Behen ME, Chugani HT, Chugani DC. In vivo detection of reduced Purkinje cell fibers with diffusion MRI tractography in children with autistic spectrum disorders. Front Hum Neurosci. 2014;8:110. doi: 10.3389/fnhum.2014.00110.	Children
65	Hanaie R, Mohri I, Kagitani-Shimono K, Tachibana M, Matsuzaki J, Watanabe Y, Fujita N, Taniike M. Abnormal corpus callosum connectivity, socio-communicative deficits, and motor deficits in children with autism spectrum disorder: a diffusion tensor imaging study. J Autism Dev Disord. 2014;44(9):2209-20. doi: 10.1007/s10803-014-2096-8.	Children
66	Cooper M, Thapar A, Jones DK. White matter microstructure predicts autistic traits in attention-deficit/hyperactivity disorder. J Autism Dev Disord. 2014;44(11):2742-54. doi: 10.1007/s10803-014-2131-9. Erratum in: J Autism Dev Disord. 2014;44(11):2755.	No ASD
67	Mevel K, Fransson P, Bölte S. Multimodal brain imaging in autism spectrum disorder and the promise of twin research. Autism. 2015;19(5):527-41. doi: 10.1177/1362361314535510. Epub 2014 Jun 10.	Review
68	Ingalhalikar M, Parker WA, Bloy L, Roberts TP, Verma R. Creating multimodal predictors using missing data: classifying and subtyping autism spectrum disorder. J Neurosci Methods. 2014;235:1-9. doi: 10.1016/j.jneumeth.2014.06.030. Epub 2014 Jun 28.	Unfocused
69	Pryweller JR, Schauder KB, Anderson AW, Heacock JL, Foss-Feig JH, Newsom CR, Loring WA, Cascio CJ. White matter correlates of sensory processing in autism spectrum disorders. Neuroimage Clin. 2014;6:379-87. doi: 10.1016/j.nicl.2014.09.018.	Children
70	Ameis SH, Catani M. Altered white matter connectivity as a neural substrate for social impairment in Autism Spectrum Disorder. Cortex. 2015;62:158-81. doi: 10.1016/j.cortex.2014.10.014. Epub 2014 Nov 5.	Review
**71**	**Itahashi T, Yamada T, Nakamura M, Watanabe H, Yamagata B, Jimbo D, Shioda S, Kuroda M, Toriizuka K, Kato N, Hashimoto R. Linked alterations in gray and white matter morphology in adults with high-functioning autism spectrum disorder: a multimodal brain imaging study. Neuroimage Clin. 2015;7:155-69. doi: 10.1016/j.nicl.2014.11.019.**	**Included**
**72**	**Libero LE, DeRamus TP, Lahti AC, Deshpande G, Kana RK. Multimodal neuroimaging based classification of autism spectrum disorder using anatomical, neurochemical, and white matter correlates. Cortex. 2015;66:46-59. doi: 10.1016/j.cortex.2015.02.008. Epub 2015 Mar 3.**	**Included**
73	DeRamus TP, Kana RK. Anatomical likelihood estimation meta-analysis of grey and white matter anomalies in autism spectrum disorders. Neuroimage Clin. 2014;7:525-36. doi: 10.1016/j.nicl.2014.11.004.	Review
74	Crawford JD, Chandley MJ, Szebeni K, Szebeni A, Waters B, Ordway GA. Elevated GFAP protein in anterior cingulate cortical white matter in males with autism spectrum disorder. Autism Res. 2015;8(6):649-57. doi: 10.1002/aur.1480. Epub 2015 Apr 6.	Post mortem
75	Roine U, Salmi J, Roine T, Wendt TN, Leppämäki S, Rintahaka P, Tani P, Leemans A, Sams M. Constrained spherical deconvolution-based tractography and tract-based spatial statistics show abnormal microstructural organization in Asperger syndrome. Mol Autism. 2015;6:4. doi: 10.1186/2040-2392-6-4.	Overlap
**76**	**Kirkovski M, Enticott PG, Maller JJ, Rossell SL, Fitzgerald PB. Diffusion tensor imaging reveals no white matter impairments among adults with autism spectrum disorder. Psychiatry Res. 2015;233(1):64-72. doi: 10.1016/j.pscychresns.2015.05.003. Epub 2015 May 14.**	**Included**
77	Ogur T, Boyunaga OL. Relation of behavior problems with findings of cranial diffusion tensor MRI and MR spectroscopy in autistic children. Int J Clin Exp Med. 2015;8(4):5621-30.	Children
78	Solso S, Xu R, Proudfoot J, Hagler DJ Jr, Campbell K, Venkatraman V, Carter Barnes C, Ahrens-Barbeau C, Pierce K, Dale A, Eyler L, Courchesne E. Diffusion tensor imaging provides evidence of possible axonal overconnectivity in frontal lobes in autism spectrum disorder toddlers. Biol Psychiatry. 2016;79(8):676-84. doi: 10.1016/j.biopsych.2015.06.029. Epub 2015 Jul 4.	Children

79	Jin Y, Wee CY, Shi F, Thung KH, Ni D, Yap PT, Shen D. Identification of infants at high-risk for autism spectrum disorder using multiparameter multiscale white matter connectivity networks. Hum Brain Mapp. 2015;36(12):4880-96. doi: 10.1002/hbm.22957. Epub 2015 Sep 14.	No DTI
80	Bennett MR, Lagopoulos J. Neurodevelopmental sequelae associated with gray and white matter changes and their cellular basis: A comparison between Autism Spectrum Disorder, ADHD and dyslexia. Int J Dev Neurosci. 2015;46:132-43. doi: 10.1016/j.ijdevneu.2015.02.007. Epub 2015 Aug 11.	No DTI
81	Blackmon K, Ben-Avi E, Wang X, Pardoe HR, Di Martino A, Halgren E, Devinsky O, Thesen T, Kuzniecky R. Periventricular white matter abnormalities and restricted repetitive behavior in autism spectrum disorder. Neuroimage Clin. 2015;10:36-45. doi: 10.1016/j.nicl.2015.10.017.	No DTI
82	Hanaie R, Mohri I, Kagitani-Shimono K, Tachibana M, Matsuzaki J, Hirata I, Nagatani F, Watanabe Y, Fujita N, Taniike M. White matter volume in the brainstem and inferior parietal lobule is related to motor performance in children with autism spectrum disorder: A voxel-based morphometry study. Autism Res. 2016;9(9):981-92. doi: 10.1002/aur.1605. Epub 2016 Jan 25.	No DTI
83	Vogan VM, Morgan BR, Leung RC, Anagnostou E, Doyle-Thomas K, Taylor MJ. Widespread white matter differences in children and adolescents with autism spectrum disorder. J Autism Dev Disord. 2016;46(6):2138-2147. doi: 10.1007/s10803-016-2744-2.	Children
84	Bohland JW. Toward a multimodal, multiscale understanding of white matter abnormalities in autism spectrum disorder. Biol Psychiatry. 2016;79(8):e47-8. doi: 10.1016/j.biopsych.2016.02.020. Epub 2016 Feb 23.	Opinion
85	Dean DC III, Travers BG, Adluru N, Tromp do PM, Destiche DJ, Samsin D, Prigge MB, Zielinski BA, Fletcher PT, Anderson JS, Froehlich AL, Bigler ED, Lange N, Lainhart JE, Alexander AL. Investigating the microstructural correlation of white matter in autism spectrum disorder. Brain Connect. 2016;6(5):415-33. doi: 10.1089/brain.2015.0385. Epub 2016 May 5.	Lumping
86	Berman JI, Edgar JC, Blaskey L, Kuschner ES, Levy SE, Ku M, Dell J, Roberts TP. Multimodal diffusion-MRI and MEG assessment of auditory and language system development in autism spectrum disorder. Front Neuroanat. 2016;10:30. doi: 10.3389/fnana.2016.00030.	Children
87	Katz J, d'Albis MA, Boisgontier J, Poupon C, Mangin JF, Guevara P, Duclap D, Hamdani N, Petit J, Monnet D, Le Corvoisier P, Leboyer M, Delorme R, Houenou J. Similar white matter but opposite grey matter changes in schizophrenia and high-functioning autism. Acta Psychiatr Scand. 2016;134(1):31-9. doi: 10.1111/acps.12579. Epub 2016 Apr 22.	No DTI
**88**	**Ecker C, Andrews D, Dell'Acqua F, Daly E, Murphy C, Catani M, Thiebaut de Schotten M, Baron-Cohen S, Lai MC, Lombardo MV, Bullmore ET, Suckling J, Williams S, Jones DK, Chiocchetti A; MRC AIMS Consortium; Murphy DG. Relationship between cortical gyrification, white matter connectivity, and autism spectrum disorder. Cereb Cortex. 2016;26(7):3297-309. doi: 10.1093/cercor/bhw098. Epub 2016 Apr 29.**	**Included**
89	Fitzgerald J, Gallagher L, McGrath J. Widespread disrupted white matter microstructure in autism spectrum disorders. J Autism Dev Disord. 2019;49(7):2664-2674. doi: 10.1007/s10803-016-2803-8.	Lumping
90	Wilkinson M, Wang R, van der Kouwe A, Takahashi E. White and gray matter fiber pathways in autism spectrum disorder revealed by ex vivo diffusion MR tractography. Brain Behav. 2016;6(7):e00483. doi: 10.1002/brb3.483.	Case
91	Ameis SH, Lerch JP, Taylor MJ, Lee W, Viviano JD, Pipitone J, Nazeri A, Croarkin PE, Voineskos AN, Lai MC, Crosbie J, Brian J, Soreni N, Schachar R, Szatmari P, Arnold PD, Anagnostou E. A diffusion tensor imaging study in children with ADHD, autism spectrum disorder, OCD, and matched controls: Distinct and non-distinct white matter disruption and dimensional brain-behavior relationships. Am J Psychiatry. 2016;173(12):1213-1222. doi: 10.1176/appi.ajp.2016.15111435. Epub 2016 Jul 1.	Children
92	Samson AC, Dougherty RF, Lee IA, Phillips JM, Gross JJ, Hardan AY. White matter structure in the uncinate fasciculus: Implications for socio-affective deficits in Autism Spectrum Disorder. Psychiatry Res Neuroimaging. 2016;255:66-74. doi: 10.1016/j.pscychresns.2016.08.004. Epub 2016 Aug 13.	Children
**93**	**Libero LE, Burge WK, Deshpande HD, Pestilli F, Kana RK. White Matter Diffusion of major fiber tracts implicated in autism spectrum disorder. Brain Connect. 2016;6(9):691-699. doi: 10.1089/brain.2016.0442. Epub 2016 Sep 30.**	**Included**
94	Chiang HL, Chen YJ, Lin HY, Tseng WI, Gau SS. Disorder-specific alteration in white matter structural property in adults with autism spectrum disorder relative to adults with ADHD and adult controls. Hum Brain Mapp. 2017;38(1):384-395. doi: 10.1002/hbm.23367. Epub 2016 Sep 15. Trial registration: ClinicalTrials.gov NCT01582256 NCT01247610.	No DTI
95	Bradstreet LE, Hecht EE, King TZ, Turner JL, Robins DL. Associations between autistic traits and fractional anisotropy values in white matter tracts in a nonclinical sample of young adults. Exp Brain Res. 2017;235(1):259-267. doi: 10.1007/s00221-016-4791-5. Epub 2016 Oct 3.	No ASD
96	Carper RA, Treiber JM, DeJesus SY, Müller RA. Reduced hemispheric asymmetry of white matter microstructure in autism spectrum disorder. J Am Acad Child Adolesc Psychiatry. 2016;55(12):1073-1080. doi: 10.1016/j.jaac.2016.09.491. Epub 2016 Sep 28.	Children
97	Mitelman SA, Bralet MC, Haznedar MM, Hollander E, Shihabuddin L, Hazlett EA, Buchsbaum MS. Diametrical relationship between gray and white matter volumes in autism spectrum disorder and schizophrenia. Brain Imaging Behav. 2017;11(6):1823-1835. doi: 10.1007/s11682-016-9648-9.	No DTI
98	Carlisi CO, Norman LJ, Lukito SS, Radua J, Mataix-Cols D, Rubia K. Comparative multimodal meta-analysis of structural and functional brain abnormalities in autism spectrum disorder and obsessive-compulsive disorder. Biol Psychiatry. 2017;82(2):83-102. doi: 10.1016/j.biopsych.2016.10.006. Epub 2016 Oct 13.	Review
99	Andrews DS, Avino TA, Gudbrandsen M, Daly E, Marquand A, Murphy CM, Lai MC, Lombardo MV, Ruigrok AN, Williams SC, Bullmore ET, The Mrc Aims Consortium, Suckling J, Baron-Cohen S, Craig MC, Murphy DG, Ecker C. In vivo evidence of reduced integrity of the gray-white matter boundary in autism spectrum disorder. Cereb Cortex. 2017;27(2):877-887. doi: 10.1093/cercor/bhw404.	No DTI
100	Dean DC III, Lange N, Travers BG, Prigge MB, Matsunami N, Kellett KA, Freeman A, Kane KL, Adluru N, Tromp DP, Destiche DJ, Samsin D, Zielinski BA, Fletcher PT, Anderson JS, Froehlich AL, Leppert MF, Bigler ED, Lainhart JE, Alexander AL. Multivariate characterization of white matter heterogeneity in autism spectrum disorder. Neuroimage Clin. 2017;14:54-66. doi: 10.1016/j.nicl.2017.01.002.	Lumping
101	Blanken LME, Muetzel RL, Jaddoe VWV, Verhulst FC, van der Lugt A, Tiemeier H, White T. White matter microstructure in children with autistic traits. Psychiatry Res Neuroimaging. 2017;263:127-134. doi: 10.1016/j.pscychresns.2017.03.015. Epub 2017 Mar 28.	Children
102	Irimia A, Torgerson CM, Jacokes ZJ, Van Horn JD. The connectomes of males and females with autism spectrum disorder have significantly different white matter connectivity densities. Sci Rep. 2017;7:46401. doi: 10.1038/srep46401.	Unfocused
**103**	**Nickel K, Tebartz van Elst L, Perlov E, Endres D, Müller GT, Riedel A, Fangmeier T, Maier S. Altered white matter integrity in adults with autism spectrum disorder and an IQ>100: a diffusion tensor imaging study. Acta Psychiatr Scand. 2017;135(6):573-583. doi: 10.1111/acps.12731. Epub 2017 Apr 13.**	**Included**
104	Chenausky K, Kernbach J, Norton A, Schlaug G. White matter integrity and treatment-based change in speech performance in minimally verbal children with autism spectrum disorder. Front Hum Neurosci. 2017;11:175. doi: 10.3389/fnhum.2017.00175.	Children
105	Martínez K, Merchán-Naranjo J, Pina-Camacho L, Alemán-Gómez Y, Boada L, Fraguas D, Moreno C, Arango C, Janssen J, Parellada M. Atypical age-dependency of executive function and white matter microstructure in children and adolescents with autism spectrum disorders. Eur Child Adolesc Psychiatry. 2017;26(11):1361-1376. doi: 10.1007/s00787-017-0990-2. Epub 2017 Apr 26.	Children
106	Li Y, Fang H, Zheng W, Qian L, Xiao Y, Wu Q, Chang C, Xiao C, Chu K, Ke X. A fiber tractography study of social-emotional related fiber tracts in children and adolescents with autism spectrum disorder. Neurosci Bull. 2017;33(6):722-730. doi: 10.1007/s12264-017-0155-9. Epub 2017 Jul 10.	Children
107	Fitzgerald J, Leemans A, Kehoe E, O'Hanlon E, Gallagher L, McGrath J. Abnormal fronto-parietal white matter organisation in the superior longitudinal fasciculus branches in autism spectrum disorders. Eur J Neurosci. 2018;47(6):652-661. doi: 10.1111/ejn.13655. Epub 2017 Sep 1.	Lumping
108	Tanaka H, Negoro H, Iwasaka H, Nakamura S. Embodied conversational agents for multimodal automated social skills training in people with autism spectrum disorders. PLoS One. 2017;12(8):e0182151. doi: 10.1371/journal.pone.0182151.	No DTI
109	Aoki Y, Yoncheva YN, Chen B, Nath T, Sharp D, Lazar M, Velasco P, Milham MP, Di Martino A. Association of white matter structure with autism spectrum disorder and attention-deficit/hyperactivity disorder. JAMA Psychiatry. 2017;74(11):1120-1128. doi: 10.1001/jamapsychiatry.2017.2573.	Children
110	Chien YL, Chen YJ, Hsu YC, Tseng WI, Gau SS. Altered white-matter integrity in unaffected siblings of probands with autism spectrum disorders. Hum Brain Mapp. 2017;38(12):6053-6067. doi: 10.1002/hbm.23810. Epub 2017 Sep 20.	No DTI
111	Ness SL, Manyakov NV, Bangerter A, Lewin D, Jagannatha S, Boice M, Skalkin A, Dawson G, Janvier YM, Goodwin MS, Hendren R, Leventhal B, Shic F, Cioccia W, Pandina G. JAKE® Multimodal Data Capture System: Insights from an observational study of autism spectrum disorder. Front Neurosci. 2017;11:517. doi: 10.3389/fnins.2017.00517.	Unfocused
112	Di X, Azeez A, Li X, Haque E, Biswal BB. Disrupted focal white matter integrity in autism spectrum disorder: A voxel-based meta-analysis of diffusion tensor imaging studies. Prog Neuropsychopharmacol Biol Psychiatry. 2018;82:242-248. doi: 10.1016/j.pnpbp.2017.11.007. Epub 2017 Nov 9.	Review
113	Mitelman SA, Buchsbaum MS, Young DS, Haznedar MM, Hollander E, Shihabuddin L, Hazlett EA, Bralet MC. Increased white matter metabolic rates in autism spectrum disorder and schizophrenia. Brain Imaging Behav. 2018;12(5):1290-1305. doi: 10.1007/s11682-017-9785-9.	No DTI
114	Wei L, Zhong S, Nie S, Gong G. Aberrant development of the asymmetry between hemispheric brain white matter networks in autism spectrum disorder. Eur Neuropsychopharmacol. 2018;28(1):48-62. doi: 10.1016/j.euroneuro.2017.11.018. Epub 2017 Dec 7.	No DTI
115	Mash LE, Reiter MA, Linke AC, Townsend J, Müller RA. Multimodal approaches to functional connectivity in autism spectrum disorders: An integrative perspective. Dev Neurobiol. 2018;78(5):456-473. doi: 10.1002/dneu.22570. Epub 2017 Dec 27.	Review

116	Litchke LG, Liu T, Castro S. Effects of multimodal Mandala Yoga on social and emotional skills for youth with Autism Spectrum Disorder: An exploratory study. Int J Yoga. 2018;11(1):59-65. doi: 10.4103/ijoy.IJOY_80_16.	Unrelated
117	Bitar T, Mavel S, Emond P, Nadal-Desbarats L, Lefèvre A, Mattar H, Soufia M, Blasco H, Vourc'h P, Hleihel W, Andres CR. Identification of metabolic pathway disturbances using multimodal metabolomics in autistic disorders in a Middle Eastern population. J Pharm Biomed Anal. 2018;152:57-65. doi: 10.1016/j.jpba.2018.01.007. Epub 2018 Feb 3.	No DTI
118	Li SJ, Wang Y, Qian L, Liu G, Liu SF, Zou LP, Zhang JS, Hu N, Chen XQ, Yu SY, Guo SL, Li K, He MW, Wu HT, Qiu JX, Zhang L, Wang YL, Lou X, Ma L. Alterations of white matter connectivity in preschool children with autism spectrum disorder. Radiology. 2018;288(1):209-217. doi: 10.1148/radiol.2018170059. Epub 2018 Mar 27.	Children
119	Kuno M, Hirano Y, Nakagawa A, Asano K, Oshima F, Nagaoka S, Matsumoto K, Masuda Y, Iyo M, Shimizu E. White matter features associated with autistic traits in obsessive-compulsive disorder. Front Psychiatry. 2018;9:216. doi: 10.3389/fpsyt.2018.00216.	No ASD
120	Karahanoğlu FI, Baran B, Nguyen QTH, Meskaldji DE, Yendiki A, Vangel M, Santangelo SL, Manoach DS. Diffusion-weighted imaging evidence of altered white matter development from late childhood to early adulthood in Autism Spectrum Disorder. Neuroimage Clin. 2018;19:840-847. doi: 10.1016/j.nicl.2018.06.002.	No DTI
121	Qin B, Wang L, Zhang Y, Cai J, Chen J, Li T. Enhanced topological network efficiency in preschool autism spectrum disorder: A diffusion tensor imaging study. Front Psychiatry. 2018;9:278. doi: 10.3389/fpsyt.2018.00278. Erratum in: Front Psychiatry. 2019;10:68. doi: 10.3389/fpsyt.2019.00068.	Children
122	Yang Q, Huang P, Li C, Fang P, Zhao N, Nan J, Wang B, Gao W, Cui LB. Mapping alterations of gray matter volume and white matter integrity in children with autism spectrum disorder: evidence from fMRI findings. Neuroreport. 2018;29(14):1188-1192. doi: 10.1097/WNR.0000000000001094.	Children
123	Qian L, Wang Y, Chu K, Li Y, Xiao C, Xiao T, Xiao X, Qiu T, Xiao Y, Fang H, Ke X. Alterations in hub organization in the white matter structural network in toddlers with autism spectrum disorder: A 2-year follow-up study. Autism Res. 2018;11(9):1218-1228. doi: 10.1002/aur.1983. Epub 2018 Aug 16.	Children
**124**	**Yamagata B, Itahashi T, Nakamura M, Mimura M, Hashimoto RI, Kato N, Aoki Y. White matter endophenotypes and correlates for the clinical diagnosis of autism spectrum disorder. Soc Cogn Affect Neurosci. 2018;13(7):765-773. doi: 10.1093/scan/nsy048.**	**Included**
125	Mann C, Bletsch A, Andrews D, Daly E, Murphy C; MRC AIMS Consortium; Murphy D, Ecker C. The effect of age on vertex-based measures of the grey-white matter tissue contrast in autism spectrum disorder. Mol Autism. 2018;9:49. doi: 10.1186/s13229-018-0232-6.	No DTI
126	Lin CW, Lin HY, Lo YC, Chen YJ, Hsu YC, Chen YL, Tseng WI, Gau SS. Alterations in white matter microstructure and regional volume are related to motor functions in boys with autism spectrum disorder. Prog Neuropsychopharmacol Biol Psychiatry. 2019;90:76-83. doi: 10.1016/j.pnpbp.2018.11.008. Epub 2018 Nov 16.	No DTI
127	Payabvash S, Palacios EM, Owen JP, Wang MB, Tavassoli T, Gerdes M, Brandes- Aitken A, Cuneo D, Marco EJ, Mukherjee P. White matter connectome edge density in children with autism spectrum disorders: Potential imaging biomarkers using machine-learning models. Brain Connect. 2019;9(2):209-220. doi: 10.1089/brain.2018.0658.	Children
128	Dimond D, Schuetze M, Smith RE, Dhollander T, Cho I, Vinette S, Ten Eycke K, Lebel C, McCrimmon A, Dewey D, Connelly A, Bray S. Reduced white matter fiber density in autism spectrum disorder. Cereb Cortex. 2019;29(4):1778-1788. doi: 10.1093/cercor/bhy348.	No DTI
129	Qin B, Wang L, Zhang Y, Cai J, Chen J, Li T. Corrigendum Enhanced topological network efficiency in preschool autism spectrum disorder: A diffusion tensor imaging study. Front Psychiatry. 2019;10:68. doi: 10.3389/fpsyt.2019.00068. Erratum for: Front Psychiatry. 2018;9:278. doi: 10.3389/fpsyt.2018.00278.	Duplicate #102
130	Smith BA, Tuduri E, Mostovoy E, Pannell D, Landon C. Evaluation of the rhythmic arts project, a multi-modal rhythm-based perception and action intervention, in a school-based setting in children with autism spectrum disorders. J Am Acad Spec Educ Prof. 2019;2019:171-179.	Unrelated
131	Kato Y, Kagitani-Shimono K, Matsuzaki J, Hanaie R, Yamamoto T, Tominaga K, Watanabe Y, Mohri I, Taniike M. White matter tract-cognitive relationships in children with high-functioning autism spectrum disorder. Psychiatry Investig. 2019;16(3):220-233. doi: 10.30773/pi.2019.01.16. Epub 2019 Mar 21.	Children
132	Zhang L, Ma R, Yuan Y, Lian D, Qi X, Zheng N, Li K. The value of diffusion tensor imaging for differentiating autism spectrum disorder with language delay from developmental language disorder among toddlers. Medicine (Baltimore). 2019;98(14):e15058. doi: 10.1097/MD.0000000000015058. Erratum in: Medicine (Baltimore). 2020;99(51):e23796. doi: 10.1097/MD.0000000000023796.	Children
133	Sokol DK, Maloney B, Westmark CJ, Lahiri DK. Novel contribution of secreted amyloid-β precursor protein to white matter brain enlargement in autism spectrum disorder. Front Psychiatry. 2019;10:165. doi: 10.3389/fpsyt.2019.00165.	Review
**134**	**Hattori A, Kamagata K, Kirino E, Andica C, Tanaka S, Hagiwara A, Fujita S, Maekawa T, Irie R, Kumamaru KK, Suzuki M, Wada A, Hori M, Aoki S. White matter alterations in adult with autism spectrum disorder evaluated using diffusion kurtosis imaging. Neuroradiology. 2019;61(12):1343-1353. doi: 10.1007/s00234-019-02238-5. Epub 2019 Jun 18.**	**Included**
**135**	**Yassin W, Kojima M, Owada K, Kuwabara H, Gonoi W, Aoki Y, Takao H, Natsubori T, Iwashiro N, Kasai K, Kano Y, Abe O, Yamasue H. Paternal age contribution to brain white matter aberrations in autism spectrum disorder. Psychiatry Clin Neurosci. 2019;73(10):649-659. doi: 10.1111/pcn.12909. Epub 2019 Jul 30.**	**Included**
**136**	**Mohajer B, Masoudi M, Ashrafi A, Mohammadi E, Bayani Ershadi AS, Aarabi MH, Uban KA. Structural white matter alterations in male adults with high functioning autism spectrum disorder and concurrent depressive symptoms; a diffusion tensor imaging study. J Affect Disord. 2019;259:40-46. doi: 10.1016/j.jad.2019.08.010. Epub 2019 Aug 13.**	**Included**
137	Saaybi S, AlArab N, Hannoun S, Saade M, Tutunji R, Zeeni C, Shbarou R, Hourani R, Boustany RM. Pre- and post-therapy assessment of clinical outcomes and white matter integrity in autism spectrum disorder: Pilot study. Front Neurol. 2019;10:877. doi: 10.3389/fneur.2019.00877.	Children
138	Haebich KM, Pride NA, Walsh KS, Chisholm A, Rouel M, Maier A, Anderson V, Barton B, Silk T, Korgaonkar M, Seal M, Lami F, Lorenzo J, Williams K, Dabscheck G, Rae CD, Kean M, North KN, Payne JM. Understanding autism spectrum disorder and social functioning in children with neurofibromatosis type 1: protocol for a cross-sectional multimodal study. BMJ Open. 2019;9(9):e030601. doi: 10.1136/bmjopen-2019-030601.	Protocol
139	Hrdlicka M, Sanda J, Urbanek T, Kudr M, Dudova I, Kickova S, Pospisilova L, Mohaplova M, Maulisova A, Krsek P, Kyncl M, Blatny M, Komarek V. Diffusion tensor imaging and tractography in autistic, dysphasic, and healthy control children. Neuropsychiatr Dis Treat. 2019;15:2843-2852. doi: 10.2147/NDT.S219545.	Children
140	Prohl AK, Scherrer B, Tomas-Fernandez X, Davis PE, Filip-Dhima R, Prabhu SP, Peters JM, Bebin EM, Krueger DA, Northrup H, Wu JY, Sahin M, Warfield SK; TACERN Study Group. Early white matter development is abnormal in tuberous sclerosis complex patients who develop autism spectrum disorder. J Neurodev Disord. 2019;11(1):36. doi: 10.1186/s11689-019-9293-x.	Children
141	Cavinato L, Cardinaux A, Jain K, Jamal W, Kjelgaard M, Sinha P, Barbieri R. Characterizing autonomic response to arousing visual-auditory multi-modal task in Autism Spectrum Disorder (ASD). Annu Int Conf IEEE Eng Med Biol Soc. 2019 Jul;2019:4942-4945. doi: 10.1109/EMBC.2019.8856641.	No DTI
**142**	**Haigh SM, Keller TA, Minshew NJ, Eack SM. Reduced white matter integrity and deficits in neuropsychological functioning in adults with autism spectrum disorder. Autism Res. 2020;13(5):702-714. doi: 10.1002/aur.2271. Epub 2020 Feb 19.**	**Included**
143	Pejhan S, Siu VM, Ang LC, Del Bigio MR, Rastegar M. Differential brain region-specific expression of MeCP2 and BDNF in Rett Syndrome patients: a distinct grey-white matter variation. Neuropathol Appl Neurobiol. 2020;46(7):735-750. doi: 10.1111/nan.12619. Epub 2020 Apr 20.	*Post-mortem*
144	Thompson A, Shahidiani A, Fritz A, O'Muircheartaigh J, Walker L, D'Almeida V, Murphy C, Daly E, Murphy D, Williams S, Deoni S, Ecker C. Age-related differences in white matter diffusion measures in autism spectrum condition. Mol Autism. 2020;11(1):36. doi: 10.1186/s13229-020-00325-6.	Children
145	Tang M, Kumar P, Chen H, Shrivastava A. Deep multimodal learning for the diagnosis of autism spectrum disorder. J Imaging. 2020;6(6):47. doi: 10.3390/jimaging6060047.	No DTI
146	Murillo E, Camacho L, Montero I. Multimodal communication in children with autism spectrum disorder and different linguistic development. J Autism Dev Disord. 2021;51(5):1528-1539. doi: 10.1007/s10803-020-04637-7.	Unrelated
147	Ni HC, Lin HY, Tseng WI, Gau SS. Association of self-regulation with white matter correlates in boys with and without autism spectrum disorder. Sci Rep. 2020;10(1):13811. doi: 10.1038/s41598-020-70836-y.	No DTI
148	Kirkovski M, Fuelscher I, Hyde C, Donaldson PH, Ford TC, Rossell SL, Fitzgerald PB, Enticott PG. Fixel based analysis reveals atypical white matter micro- and macrostructure in adults with autism spectrum disorder: An investigation of the role of biological sex. Front Integr Neurosci. 2020;14:40. doi: 10.3389/fnint.2020.00040.	No DTI
149	Roberts TPL, Bloy L, Ku M, Blaskey L, Jackel CR, Edgar JC, Berman JI. A multimodal study of the contributions of conduction velocity to the auditory evoked neuromagnetic response: Anomalies in autism spectrum disorder. Autism Res. 2020;13(10):1730-1745. doi: 10.1002/aur.2369. Epub 2020 Sep 14.	Children
150	Stephens K, Silk TJ, Anderson V, Hazell P, Enticott PG, Sciberras E. Associations between limbic system white matter structure and socio-emotional functioning in children with ADHD + ASD. J Autism Dev Disord. 2021;51(8):2663-2672. doi: 10.1007/s10803-020-04738-3.	No DTI
**151**	**Ohta H, Aoki YY, Itahashi T, Kanai C, Fujino J, Nakamura M, Kato N, Hashimoto RI. White matter alterations in autism spectrum disorder and attention-deficit/hyperactivity disorder in relation to sensory profile. Mol Autism. 2020;11(1):77. doi: 10.1186/s13229-020-00379-6.**	**Included**

**152**	**Bletsch A, Schäfer T, Mann C, Andrews DS, Daly E, Gudbrandsen M, Ruigrok ANV, Dallyn R, Romero-Garcia R, Lai MC, Lombardo MV, Craig MC, Suckling J, Bullmore ET, Baron-Cohen S; MRC AIMS Consortium; Murphy DGM, Dell'Acqua F, Ecker C. Atypical measures of diffusion at the gray-white matter boundary in autism spectrum disorder in adulthood. Hum Brain Mapp. 2021;42(2):467-484. doi: 10.1002/hbm.25237. Epub 2020 Oct 23.**	**Included**
153	Chen H, Long J, Yang S, He B. Atypical functional covariance connectivity between gray and white matter in children with autism spectrum disorder. Autism Res. 2021;14(3):464-472. doi: 10.1002/aur.2435. Epub 2020 Nov 18.	No DTI
154	Qi S, Morris R, Turner JA, Fu Z, Jiang R, Deramus TP, Zhi D, Calhoun VD, Sui J. Common and unique multimodal covarying patterns in autism spectrum disorder subtypes. Mol Autism. 2020;11(1):90. doi: 10.1186/s13229-020-00397-4.	No DTI
155	The value of diffusion tensor imaging for differentiating autism spectrum disorder with language delay from developmental language disorder among toddlers: Erratum. Medicine (Baltimore). 2020 Dec 18;99(51):e23796. doi: 10.1097/MD.0000000000023796. Erratum for: Medicine (Baltimore). 2019;98(14):e15058. doi: 10.1097/MD.0000000000015058.	Duplicate #113
156	Zheng ZK, Staubitz JE, Weitlauf AS, Staubitz J, Pollack M, Shibley L, Hopton M, Martin W, Swanson A, Juárez P, Warren ZE, Sarkar N. A predictive multimodal framework to alert caregivers of problem behaviors for children with ASD (PreMAC). Sensors (Basel). 2021;21(2):370. doi: 10.3390/s21020370.	Unrelated
157	Schröder Y, Hohmann DM, Meller T, Evermann U, Pfarr JK, Jansen A, Kamp- Becker I, Grezellschak S, Nenadić I. Associations of subclinical autistic-like traits with brain structural variation using diffusion tensor imaging and voxel- based morphometry. Eur Psychiatry. 2021;64(1):e27. doi: 10.1192/j.eurpsy.2021.15.	No ASD
158	Li C, Li Y, Fu L, Wang Y, Cheng X, Cui X, Jiang J, Xiao T, Ke X, Fang H. The relationships between the topological properties of the whole-brain white matter network and the severity of autism spectrum disorder: A study from monozygotic twins. Neuroscience. 2021;465:60-70. doi: 10.1016/j.neuroscience.2021.04.003. Epub 2021 Apr 20.	Children
159	Walsh MJM, Wallace GL, Gallegos SM, Braden BB. Brain-based sex differences in autism spectrum disorder across the lifespan: A systematic review of structural MRI, fMRI, and DTI findings. Neuroimage Clin. 2021;31:102719. doi: 10.1016/j.nicl.2021.102719. Epub 2021 Jun 9.	Review
160	Godel M, Andrews DS, Amaral DG, Ozonoff S, Young GS, Lee JK, Wu Nordahl C, Schaer M. Altered gray-white matter boundary contrast in toddlers at risk for autism relates to later diagnosis of autism spectrum disorder. Front Neurosci. 2021;15:669194. doi: 10.3389/fnins.2021.669194.	No ASD
161	Prats C, Fatjó-Vilas M, Penzol MJ, Kebir O, Pina-Camacho L, Demontis D, Crespo-Facorro B, Peralta V, González-Pinto A, Pomarol-Clotet E, Papiol S, Parellada M, Krebs MO, Fañanás L. Association and epistatic analysis of white matter related genes across the continuum schizophrenia and autism spectrum disorders: The joint effect of NRG1-ErbB genes. World J Biol Psychiatry. 2022;23(3):208-218. doi: 10.1080/15622975.2021.1939155. Epub 2021 Aug 2.	No DTI
162	Peterson BS, Liu J, Dantec L, Newman C, Sawardekar S, Goh S, Bansal R. Using tissue microstructure and multimodal MRI to parse the phenotypic heterogeneity and cellular basis of autism spectrum disorder. J Child Psychol Psychiatry. 2022;63(8):855-870. doi: 10.1111/jcpp.13531. Epub 2021 Nov 11.	Lumping
163	Zhao Y, Yang L, Gong G, Cao Q, Liu J. Identify aberrant white matter microstructure in ASD, ADHD and other neurodevelopmental disorders: A meta- analysis of diffusion tensor imaging studies. Prog Neuropsychopharmacol Biol Psychiatry. 2022;113:110477. doi: 10.1016/j.pnpbp.2021.110477. Epub 2021 Nov 16.	Review
164	Hijab MHF, Al-Thani D, Banire B. A multimodal messaging App (MAAN) for adults with autism spectrum disorder: Mixed methods evaluation study. JMIR Form Res. 2021;5(12):e33123. doi: 10.2196/33123.	Unrelated
**165**	**Arunachalam Chandran V, Pliatsikas C, Neufeld J, O'Connell G, Haffey A, DeLuca V, Chakrabarti B. Brain structural correlates of autistic traits across the diagnostic divide: A grey matter and white matter microstructure study. Neuroimage Clin. 2021;32:102897. doi: 10.1016/j.nicl.2021.102897. Epub 2021 Nov 23.**	**Included**
166	ElNakieb Y, Ali MT, Elnakib A, Shalaby A, Soliman A, Mahmoud A, Ghazal M, Barnes GN, El-Baz A. The role of diffusion tensor MR imaging (DTI) of the brain in diagnosing autism spectrum disorder: Promising results. Sensors (Basel). 2021;21(24):8171. doi: 10.3390/s21248171.	Unfocused
167	Kim JI, Bang S, Yang JJ, Kwon H, Jang S, Roh S, Kim SH, Kim MJ, Lee HJ, Lee JM, Kim BN. Classification of preschoolers with low-functioning autism spectrum disorder using multimodal MRI data. J Autism Dev Disord. 2023;53(1):25-37. doi: 10.1007/s10803-021-05368-z. Epub 2022 Jan 4.	Unfocused
**168**	**Yoshikawa H, Kitamura S, Matsuoka K, Takahashi M, Ishida R, Kishimoto N, Yasuno F, Yasuda Y, Hashimoto R, Miyasaka T, Kichikawa K, Kishimoto T, Makinodan M. Adverse childhood experience is associated with disrupted white matter integrity in autism spectrum disorder: A diffusion tensor imaging study. Front Psychiatry. 2022;12:823260. doi: 10.3389/fpsyt.2021.823260.**	**Included**
169	Virues-Ortega J, McKay NS, McCormack JC, Lopez N, Liu R, Kirk I. A callosal biomarker of behavioral intervention outcomes for autism spectrum disorder? A case-control feasibility study with diffusion tensor imaging. PLoS One. 2022;17(2):e0262563. doi: 10.1371/journal.pone.0262563.	Children
170	Rigby MJ, Orefice NS, Lawton AJ, Ma M, Shapiro SL, Yi SY, Dieterich IA, Frelka A, Miles HN, Pearce RA, Yu JPJ, Li L, Denu JM, Puglielli L. SLC13A5/sodium-citrate co-transporter overexpression causes disrupted white matter integrity and an autistic-like phenotype. Brain Commun. 2022;4(1):fcac002. doi: 10.1093/braincomms/fcac002.	Animal
171	Ma L, Liu M, Xue K, Ye C, Man W, Cheng M, Liu Z, Zhu D, Liu F, Wang J. Abnormal regional spontaneous brain activities in white matter in patients with autism spectrum disorder. Neuroscience. 2022;490:1-10. doi: 10.1016/j.neuroscience.2022.02.022. Epub 2022 Feb 23.	No DTI
172	Bagherzadeh-Azbari S, Lau GKB, Ouyang G, Zhou C, Hildebrandt A, Sommer W, Lui M. Multimodal evidence of atypical processing of eye gaze and facial emotion in children with autistic traits. Front Hum Neurosci. 2022;16:733852. doi: 10.3389/fnhum.2022.733852.	Unrelated
173	Kong Y, Li QB, Yuan ZH, Jiang XF, Zhang GQ, Cheng N, Dang N. Multimodal Neuroimaging in Rett Syndrome With MECP2 Mutation. Front Neurol. 2022 Feb 23;13:838206. doi: 10.3389/fneur.2022.838206.	Review
174	Chien YL, Lin HY, Tung YH, Hwang TJ, Chen CL, Wu CS, Shang CY, Hwu HG, Tseng WI, Liu CM, Gau SS. Neurodevelopmental model of schizophrenia revisited: similarity in individual deviation and idiosyncrasy from the normative model of whole-brain white matter tracts and shared brain-cognition covariation with ADHD and ASD. Mol Psychiatry. 2022;27(8):3262-3271. doi: 10.1038/s41380-022-01636-1. Epub 2022 Jul 6.	Unfocused
175	da Cruz FM. Multimodal interaction analysis of non-lexical vocalisations in low-verbal autistic children. Clin Linguist Phon. 2023;37(4-6):491-512. doi: 10.1080/02699206.2022.2082887. Epub 2022 Jul 13.	Unrelated
176	Han J, Jiang G, Ouyang G, Li X. A multimodal approach for identifying autism spectrum disorders in children. IEEE Trans Neural Syst Rehabil Eng. 2022;30:2003-2011. doi: 10.1109/TNSRE.2022.3192431. Epub 2022 Jul 22.	No DTI
**177**	**Cai Y, Zhao J, Wang L, Xie Y, Fan X. Altered topological properties of white matter structural network in adults with autism spectrum disorder. Asian J Psychiatr. 2022;75:103211. doi: 10.1016/j.ajp.2022.103211. Epub 2022 Jul 16.**	**Included**
178	Li M, Wang Y, Tachibana M, Rahman S, Kagitani-Shimono K. Atypical structural connectivity of language networks in autism spectrum disorder: A meta-analysis of diffusion tensor imaging studies. Autism Res. 2022;15(9):1585-1602. doi: 10.1002/aur.2789. Epub 2022 Aug 13.	Review
179	Takeguchi R, Kuroda M, Tanaka R, Suzuki N, Akaba Y, Tsujimura K, Itoh M, Takahashi S. Structural and functional changes in the brains of patients with Rett syndrome: A multimodal MRI study. J Neurol Sci. 2022;441:120381. doi: 10.1016/j.jns.2022.120381. Epub 2022 Aug 18.	Children
180	Vanes LD, Tye C, Tournier JD, Combes AJE, Shephard E, Liang H, Barker GJ, Nosarti C; TS2000 Study Team; Bolton P. White matter disruptions related to inattention and autism spectrum symptoms in tuberous sclerosis complex. Neuroimage Clin. 2022;36:103163. doi: 10.1016/j.nicl.2022.103163. Epub 2022 Aug 25.	No ASD
181	Weber CF, Lake EMR, Haider SP, Mozayan A, Mukherjee P, Scheinost D, Bamford NS, Ment L, Constable T, Payabvash S. Age-dependent white matter microstructural disintegrity in autism spectrum disorder. Front Neurosci. 2022;16:957018. doi: 10.3389/fnins.2022.957018.	Lumping
182	Chien YL, Chen YJ, Tseng WL, Hsu YC, Wu CS, Tseng WI, Gau SS. Differences in white matter segments in autistic males, non-autistic siblings, and non-autistic participants: An intermediate phenotype approach. Autism. 2023;27(4):1036-1052. doi: 10.1177/13623613221125620. Epub 2022 Oct 18.	No DTI
183	Simhal AK, Carpenter KLH, Kurtzberg J, Song A, Tannenbaum A, Zhang L, Sapiro G, Dawson G. Changes in the geometry and robustness of diffusion tensor imaging networks: Secondary analysis from a randomized controlled trial of young autistic children receiving an umbilical cord blood infusion. Front Psychiatry. 2022;13:1026279. doi: 10.3389/fpsyt.2022.1026279.	Unfocused
184	Li X, Ruan C, Zibrila AI, Musa M, Wu Y, Zhang Z, Liu H, Salimeen M. Children with autism spectrum disorder present glymphatic system dysfunction evidenced by diffusion tensor imaging along the perivascular space. Medicine (Baltimore). 2022;101(48):e32061. doi: 10.1097/MD.0000000000032061.	Unfocused
185	Surgent O, Riaz A, Ausderau KK, Adluru N, Kirk GR, Guerrero-Gonzalez J, Skaletski EC, Kecskemeti SR, Dean Iii DC, Weismer SE, Alexander AL, Travers BG. Brainstem white matter microstructure is associated with hyporesponsiveness and overall sensory features in autistic children. Mol Autism. 2022;13(1):48. doi: 10.1186/s13229-022-00524-3.	Children
186	Hung Y, Dallenbach NT, Green A, Gaillard S, Capella J, Hoskova B, Vater CH, Cooper E, Rudberg N, Takahashi A, Gabrieli JDE, Joshi G. Distinct and shared white matter abnormalities when ADHD is comorbid with ASD: A preliminary diffusion tensor imaging study. Psychiatry Res. 2023;320:115039. doi: 10.1016/j.psychres.2022.115039. Epub 2022 Dec 28.	Children
187	Abbas SQ, Chi L, Chen YP. DeepMNF Deep multimodal neuroimaging framework for diagnosing autism spectrum disorder. Artif Intell Med. 2023;136:102475. doi: 10.1016/j.artmed.2022.102475. Epub 2022 Dec 21.	No DTI

188	Zhang K, Fu Z, Lai Q, Zhao Y, Liu J, Cao Q. The shared white matter developmental trajectory anomalies of attention-deficit/hyperactivity disorder and autism spectrum disorders: A meta-analysis of diffusion tensor imaging studies. Prog Neuropsychopharmacol Biol Psychiatry. 2023;124:110731. doi: 10.1016/j.pnpbp.2023.110731. Epub 2023 Feb 9.	Review
189	Zhu FL, Wang SH, Liu WB, Zhu HL, Li M, Zou XB. A multimodal machine learning system in early screening for toddlers with autism spectrum disorders based on the response to name. Front Psychiatry. 2023;14:1039293. doi: 10.3389/fpsyt.2023.1039293.	Unrelated
190	Zhou TC, Yin CG, Wang C, Shi ZF, Tong GL. Diffusion tensor imaging analysis of the corpus callosum in children with high-risk autism spectrum disorder. Technol Health Care. 2023;31(4):1451-1456. doi: 10.3233/THC-220777.	Children
191	Dehghani M, Jafarnezhadgero AA, Darvishani MA, Aali S, Granacher U. Effects of an 8-week multimodal exercise program on ground reaction forces and plantar pressure during walking in boys with autism spectrum disorder. Trials. 2023;24(1):170. doi: 10.1186/s13063-023-07158-7.	Unrelated
192	Adiani D, Breen M, Migovich M, Wade J, Hunt S, Tauseef M, Khan N, Colopietro K, Lanthier M, Swanson A, Vogus TJ, Sarkar N. Multimodal job interview simulator for training of autistic individuals. Assist Technol. 2024;36(1):22-39. doi: 10.1080/10400435.2023.2188907. Epub 2023 Apr 24.	Unrelated
193	Kirkovski M, Singh M, Dhollander T, Fuelscher I, Hyde C, Albein-Urios N, Donaldson PH, Enticott PG. An investigation of age-related neuropathophysiology in autism spectrum disorder using fixel-based analysis of corpus callosum white matter micro- and macrostructure. J Autism Dev Disord. 2024;54(6):2198-2210. doi: 10.1007/s10803-023-05980-1. Epub 2023 Apr 20.	No DTI
194	Berman JI, Bloy L, Blaskey L, Jackel CR, Miller JS, Ross J, Edgar JC, Roberts TPL. Contributions to auditory system conduction velocity: insights with multi-modal neuroimaging and machine learning in children with ASD and XYY syndrome. Front Psychiatry. 2023;14:1057221. doi: 10.3389/fpsyt.2023.1057221.	Children
195	Ratnaik R, Rakshe C, Kumar M, Agastinose Ronickom JF. Diagnostic classification of ASD improves with structural connectivity of DTI and logistic regression. Stud Health Technol Inform. 2023;305:64-67. doi: 10.3233/SHTI230425.	Children
196	Hegarty JP II, Monterrey JC, Tian Q, Cleveland SC, Gong X, Phillips JM, Wolke ON, McNab JA, Hallmayer JF, Reiss AL, Hardan AY, Lazzeroni LC. A twin study of altered white matter heritability in youth with autism spectrum disorder. J Am Acad Child Adolesc Psychiatry. 2024;63(1):65-79. doi: 10.1016/j.jaac.2023.05.030. Epub 2023 Jul 3.	Children
197	Ko C, Kang S, Hong SB, Park YR. Protocol for the development of joint attention-based subclassification of autism spectrum disorder and validation using multi-modal data. BMC Psychiatry. 2023;23(1):589. doi: 10.1186/s12888-023-04978-4.	Protocol
198	Wang M, Guo J, Wang Y, Yu M, Guo J. Multimodal autism spectrum disorder diagnosis method based on DeepGCN. IEEE Trans Neural Syst Rehabil Eng. 2023;31:3664-3674. doi: 10.1109/TNSRE.2023.3314516. Epub 2023 Sep 20.	No DTI
199	Faraji R, Ganji Z, Zamanpour SA, Nikparast F, Akbari-Lalimi H, Zare H. Impaired white matter integrity in infants and young children with autism spectrum disorder: What evidence does diffusion tensor imaging provide? Psychiatry Res Neuroimaging. 2023;335:111711. doi: 10.1016/j.pscychresns.2023.111711. Epub 2023 Aug 30.	Review
200	Ćirović M, Jeličić L, Maksimović S, Fatić S, Marisavljević M, Bošković Matić T, Subotić M. EEG Correlates of cognitive functions in a child with ASD and white matter signal abnormalities: A case report with two-and-a-half-year follow-up. Diagnostics (Basel). 2023;13(18):2878. doi: 10.3390/diagnostics13182878.	Case
**201**	**DiPiero M, Cordash H, Prigge MB, King CK, Morgan J, Guerrero-Gonzalez J, Adluru N, King JB, Lange N, Bigler ED, Zielinski BA, Alexander AL, Lainhart JE, Dean DC III. Tract- and gray matter-based spatial statistics show white matter and gray matter microstructural differences in autistic males. Front Neurosci. 2023;17:1231719. doi: 10.3389/fnins.2023.1231719.**	**Included**
202	Wang M, Xu D, Zhang L, Jiang H. Application of multimodal MRI in the early diagnosis of autism spectrum disorders: A review. Diagnostics (Basel). 2023;13(19):3027. doi: 10.3390/diagnostics13193027.	Review
203	Chen K, Zhuang W, Zhang Y, Yin S, Liu Y, Chen Y, Kang X, Ma H, Zhang T. Alteration of the large-scale white-matter functional networks in autism spectrum disorder. Cereb Cortex. 2023;33(24):11582-11593. doi: 10.1093/cercor/bhad392.	No DTI
204	Yan H. Artificial neural networks based multimodal device for autism spectrum disorder. Bratisl Lek Listy. 2023;124(11):862-869. doi: 10.4149/BLL_2023_133.	No DTI
205	Huang X, Ming Y, Zhao W, Feng R, Zhou Y, Wu L, Wang J, Xiao J, Li L, Shan X, Cao J, Kang X, Chen H, Duan X. Developmental prediction modeling based on diffusion tensor imaging uncovering age-dependent heterogeneity in early childhood autistic brain. Mol Autism. 2023;14(1):41. doi: 10.1186/s13229-023-00573-2.	Children
206	Wadhera T. Multimodal Kernel-based discriminant correlation analysis data-fusion approach: an automated autism spectrum disorder diagnostic system. Phys Eng Sci Med. 2024;47(1):361-369. doi: 10.1007/s13246-023-01350-4. Epub 2023 Nov 20.	No DTI
207	Vinçon-Leite A, Saitovitch A, Lemaître H, Rechtman E, Boisgontier J, Fillon L, Philippe A, Rio M, Desguerre I, Fabre A, Aljabali K, Boddaert N, Zilbovicius M. Identifying interindividual variability of social perception and associated brain anatomical correlations in children with autism spectrum disorder using eye-tracking and diffusion tensor imaging MRI (DTI-MRI). Cereb Cortex. 2024;34(1):bhad434. doi: 10.1093/cercor/bhad434.	Unfocused
208	Weber CF, Lake EMR, Haider SP, Mozayan A, Bobba PS, Mukherjee P, Scheinost D, Constable RT, Ment L, Payabvash S. Autism spectrum disorder-specific changes in white matter connectome edge density based on functionally defined nodes. Front Neurosci. 2023;17:1285396. doi: 10.3389/fnins.2023.1285396.	Lumping
209	Shen Y, Zhao X, Wang K, Sun Y, Zhang X, Wang C, Yang Z, Feng Z, Zhang X. Exploring white matter abnormalities in young children with autism spectrum disorder: Integrating multi-shell diffusion data and machine learning analysis. Acad Radiol. 2024;31(5):2074-2084. doi: 10.1016/j.acra.2023.12.023. Epub 2024 Jan 6.	Children
210	Wilkes BJ, Archer DB, Farmer AL, Bass C, Korah H, Vaillancourt DE, Lewis MH. Cortico-basal ganglia white matter microstructure is linked to restricted repetitive behavior in autism spectrum disorder. Mol Autism. 2024;15(1):6. doi: 10.1186/s13229-023-00581-2.	Children
211	McFayden TC, Rutsohn J, Cetin G, Forsen E, Swanson MR, Meera SS, Wolff JJ, Elison JT, Shen MD, Botteron K, Dager SR, Estes A, Gerig G, McKinstry RC, Pandey J, Schultz R, St John T, Styner M, Truong Y, Zwaigenbaum L, Hazlett HC, Piven J, Girault JB; IBIS Network. White matter development and language abilities during infancy in autism spectrum disorder. Mol Psychiatry. 2024;29(7):2095-2104. doi: 10.1038/s41380-024-02470-3. Epub 2024 Feb 21.	Children
212	Manjur SM, Diaz LRM, Lee IO, Skuse DH, Thompson DA, Marmolejos-Ramos F, Constable PA, Posada-Quintero HF. Detecting autism spectrum disorder and attention deficit hyperactivity disorder using multimodal time-frequency analysis with machine learning using the electroretinogram from two flash strengths. J Autism Dev Disord. 2024. doi: 10.1007/s10803-024-06290-w. Epub ahead of print 2024 Feb 23.	No DTI
**213**	**Weerasekera A, Ion-Mărgineanu A, Nolan GP, Mody M. Subcortical-cortical white matter connectivity in adults with autism spectrum disorder and schizophrenia patients. Psychiatry Res Neuroimaging. 2024;340:111806. doi: 10.1016/j.pscychresns.2024.111806. Epub 2024 Mar 7.**	**Included**
214	Guo Z, Tang X, Xiao S, Yan H, Sun S, Yang Z, Huang L, Chen Z, Wang Y. Systematic review and meta-analysis: multimodal functional and anatomical neural alterations in autism spectrum disorder. Mol Autism. 2024;15(1):16. doi: 10.1186/s13229-024-00593-6.	Review
215	Yi T, Ji C, Wei W, Wu G, Jin K, Jiang G. Cortical-cerebellar circuits changes in preschool ASD children by multimodal MRI. Cereb Cortex. 2024;34(4):bhae090. doi: 10.1093/cercor/bhae090.	Children
216	Wang H, Jing H, Yang J, Liu C, Hu L, Tao G, Zhao Z, Shen N. Identifying autism spectrum disorder from multi-modal data with privacy-preserving. Npj Ment Health Res. 2024;3(1):15. doi: 10.1038/s44184-023-00050-x.	No DTI
217	Srivastava S, Yang F, Prohl AK, Davis PE, Capal JK, Filip-Dhima R, Bebin EM, Krueger DA, Northrup H, Wu JY, Warfield SK, Sahin M, Zhang B; TACERN Study Group. Abnormality of early white matter development in tuberous sclerosis complex and autism spectrum disorder: Longitudinal analysis of diffusion tensor imaging measures. J Child Neurol. 2024;39(5-6):178-189. doi: 10.1177/08830738241248685. Epub 2024 May 15.	Children
218	Li M, Izumoto M, Wang Y, Kato Y, Iwatani Y, Hirata I, Mizuno Y, Tachibana M, Mohri I, Kagitani-Shimono K. Altered white matter connectivity of ventral language networks in autism spectrum disorder: An automated fiber quantification analysis with multi-site datasets. Neuroimage. 2024;297:120731. doi: 10.1016/j.neuroimage.2024.120731. Epub 2024 Jul 13.	Children
219	Feng Y, Huang X, Zhao W, Ming Y, Zhou Y, Feng R, Xiao J, Shan X, Kang X, Duan X, Chen H. Association among internalizing problems, white matter integrity, and social difficulties in children with autism spectrum disorder. Prog Neuropsychopharmacol Biol Psychiatry. 2024;135:111109. doi: 10.1016/j.pnpbp.2024.111109. Epub 2024 Jul 27.	Children
220	Benabderrahmane B, Gharzouli M, Benlecheb A. A novel multi-modal model to assist the diagnosis of autism spectrum disorder using eye-tracking data. Health Inf Sci Syst. 2024;12(1):40. doi: 10.1007/s13755-024-00299-2.	No DTI
221	Symeonides C, Vacy K, Thomson S, Tanner S, Chua HK, Dixit S, Mansell T, O'Hely M, Novakovic B, Herbstman JB, Wang S, Guo J, Chia J, Tran NT, Hwang SE, Britt K, Chen F, Kim TH, Reid CA, El-Bitar A, Bernasochi GB, Delbridge LMD, Harley VR, Yap YW, Dewey D, Love CJ, Burgner D, Tang MLK, Sly PD, Saffery R, Mueller JF, Rinehart N, Tonge B, Vuillermin P; BIS Investigator Group; Ponsonby AL, Boon WC. Male autism spectrum disorder is linked to brain aromatase disruption by prenatal BPA in multimodal investigations and 10HDA ameliorates the related mouse phenotype. Nat Commun. 2024;15(1):6367. doi: 10.1038/s41467-024-48897-8.	No DTI
222	Yang Y, Tang D, Wang Z, Liu Y, Chen F, Jie B, Ni T, Xu C, Li J, Wang C. Identification of high-functioning autism spectrum disorders based on gray-white matter functional network connectivity. J Psychiatr Res. 2024;178:107-113. doi: 10.1016/j.jpsychires.2024.08.006. Epub 2024 Aug 6.	No DTI

223	Shin YS, Christensen D, Wang J, Shirley DJ, Orlando AM, Romero RA, Wilkes BJ, Vaillancourt DE, Coombes S, Wang Z. Transcallosal white matter and cortical gray matter variations in autistic adults ages 30-73 years: A bi-tensor free water imaging approach. Res Sq [Preprint]. 2024 Aug 16:rs.3.rs-4907999. doi: 10.21203/rs.3.rs-4907999/v1.	Duplicate of #214
224	Wei L, Xu X, Su Y, Lan M, Wang S, Zhong S. Abnormal multimodal neuroimaging patterns associated with social deficits in male autism spectrum disorder. Hum Brain Mapp. 2024;45(13):e70017. doi: 10.1002/hbm.70017.	No DTI
225	Chen W, Yang J, Sun Z, Zhang X, Tao G, Ding Y, Gu J, Bu J, Wang H. DeepASD: a deep adversarial-regularized graph learning method for ASD diagnosis with multimodal data. Transl Psychiatry. 2024;14(1):375. doi: 10.1038/s41398-024-02972-2.	No DTI
226	Qing P, Zhang X, Liu Q, Huang L, Xu D, Le J, Kendrick KM, Lai H, Zhao W. Structure-function coupling in white matter uncovers the hypoconnectivity in autism spectrum disorder. Mol Autism. 2024;15(1):43. doi: 10.1186/s13229-024-00620-6.	Children
227	Zahiri J, Mirzaie M, Duan K, Xiao Y, Aamodt C, Yang X, Nazari S, Andreason C, Lopez L, Barnes CC, Arias S, Nalabolu S, Garmire L, Wang T, Hoekzema K, Eichler EE, Pierce K, Lewis NE, Courchesne E. Beyond the spectrum: Subtype- specific molecular insights into autism spectrum disorder via multimodal data integration. medRxiv [Preprint]. 2024 Sep 23:2024.09.17.24313857. doi: 10.1101/2024.09.17.24313857.	No DTI
228	Wan L, Li Y, Zhu G, Yang D, Li F, Wang W, Chen J, Yang G, Li R. Multimodal investigation of dynamic brain network alterations in autism spectrum disorder: Linking connectivity dynamics to symptoms and developmental trajectories. Neuroimage. 2024;302:120895. doi: 10.1016/j.neuroimage.2024.120895. Epub ahead of print 2024 Oct 18.	No DTI
229	Zovetti N, Meller T, Evermann U, Pfarr JK, Hoffmann J, Federspiel A, Walther S, Grezellschak S, Jansen A, Abu-Akel A, Nenadić I. Multimodal imaging of the amygdala in non-clinical subjects with high vs. low autistic-like social skills traits. Psychiatry Res Neuroimaging. 2024:111910. doi: 10.1016/j.pscychresns.2024.111910. Epub ahead of print 2024 Oct 22.	No ASD
230	Wang L, Ding S, Qin W, Zhang Y, Qin B, Huang K, Zheng H, Cai J. Alterations in the white matter fiber tracts of preschool-aged children with autism spectrum disorder: an automated fiber quantification study. Quant Imaging Med Surg. 2024;14(12):9347-9360. doi: 10.21037/qims-24-950. Epub 2024 Nov 29.	Children
231	Khan K, Katarya R. MCBERT: A multi-modal framework for the diagnosis of autism spectrum disorder. Biol Psychol. 2025;194:108976. doi: 10.1016/j.biopsycho.2024.108976. Epub 2024 Dec 23.	Review
232	Gao L, Qiao S, Zhang Y, Zhang T, Lu H, Guo X. Parsing the heterogeneity of brain structure and function in male children with autism spectrum disorder: a multimodal MRI study. Brain Imaging Behav. 2025. doi: 10.1007/s11682-025-00978-y. Epub ahead of print 2025 Feb 18.	Children
233	Wang J, Kawata NYS, Cao X, Zhang J, Fujisawa TX, Zhang X, Fan L, Xia W, Wu L, Tomoda A. White-Matter fiber tract and resting-state functional connectivity abnormalities in young children with autism spectrum disorder. Neuroimage. 2025;310:121109. doi: 10.1016/j.neuroimage.2025.121109. Epub ahead of print 2025 Feb 28.	Children
234	Gao J, Song S. A hierarchical feature extraction and multimodal deep feature integration-based model for autism spectrum disorder identification. IEEE J Biomed Health Inform. 2025;PP. doi: 10.1109/JBHI.2025.3540894. Epub ahead of print 2025 Feb 12.	No DTI
235	Huang X, Zhao W, Feng R, Zhou Y, Wang J, Xiao J, Li L, Shan X, Feng Y, Ming Y, Cao J, Kang X, Wu L, Chen H, Duan X. Linking gut microbiome profiles and white matter integrity to social behavior in young autistic children: from the perspective of individual variation. Sci Bull (Beijing). 2025:S2095-9273(25)00197-5. doi: 10.1016/j.scib.2025.02.031. Epub ahead of print 2025 Feb 25.	Children
**236**	**Shin YS, Christensen D, Wang J, Shirley DJ, Orlando AM, Romero RA, Vaillancourt DE, Wilkes BJ, Coombes SA, Wang Z. Transcallosal white matter and cortical gray matter variations in autistic adults aged 30-73 years. Mol Autism. 2025;16(1):16. doi: 10.1186/s13229-025-00652-6. Epub 2025 Mar 6.**	**Included**
237	Shen G, Green HL, McNamee M, Franzen RE, DiPiero M, Berman JI, Ku M, Bloy L, Liu S, Airey M, Goldin S, Blaskey L, Kuschner ES, Kim M, Konka K, Miller GA, Edgar JC. White matter microstructure as a potential contributor to differences in resting state alpha activity between neurotypical and autistic children: a longitudinal multimodal imaging study. Mol Autism. 2025;16(1):19. doi: 10.1186/s13229-025-00646-4. Epub 2025 Mar 11.	Children
238	Del Casale A, Shehu D, Rossi-Espagnet MC, Zocchi C, Bilotta I, Arena JF, Alcibiade A, Adriani B, Longo D, Gandolfo C, Romano A, Ferracuti S, Bozzao A, Napolitano A. Multimodal morphometric similarity network analysis of autism spectrum disorder. Brain Sci. 2025;15(3):247. doi: 10.3390/brainsci15030247.	Pooled
239	Coleman CR, Nance MG, Jacokes Z, Druzgal TJ, Arutiunian V, Kresse A, Sullivan CAW, Santhosh M, Neuhaus E, Borland H, Bernier RA, Bookheimer SY, Dapretto M, Jack A, Jeste S, McPartland JC, Naples A, Geschwind D, Gupta AR, Webb SJ, Pelphrey KA, Van Horn JD, Newman BT, Puglia MH. Structural determinants of signal speed: A multimodal investigation of face processing in autism spectrum disorder. bioRxiv [Preprint]. 2025 Mar 19:2025.03.19.644214. doi: 10.1101/2025.03.19.644214.	Children
240	Canada K, Evans TM, Pelphrey KA. Microglial regulation of white matter development and its disruption in autism spectrum disorder. Cereb Cortex. 2025;35(4):bhaf109. doi: 10.1093/cercor/bhaf109.	Review
241	Davison KE, Liu T, Belisle RM, Perrachione TK, Qi Z, Gabrieli JDE, Tager-Flusberg H, Zuk J. Right-Hemispheric White Matter Organization Is Associated With Speech Timing in Autistic Children. J Speech Lang Hear Res. 2025:1-15. doi: 10.1044/2025_JSLHR-24-00548. Epub ahead of print 2025 May 19.	Children
**PsycINFO**	TI (autism spectrum OR autistic OR ASD OR high-functioning autism OR Asperger OR Rett) AND TI (DTI OR diffusion tensor OR tractography) AND TI (adult OR adults) 21.5.2025 → 6 results	
242	Cubon, Valerie Anne. Assessment, identification, and classification of cortical abnormalities in autistic adults using diffusion tensor imaging of underlying white matter. Dissertation Abstracts International: Section B: The Sciences and Engineering, 2008	Review
243	Bloemen, Oswald J. N.; Deeley, Quinton; Sundram, Fred; et al. White matter integrity in Asperger syndrome: A preliminary diffusion tensor magnetic resonance imaging study in adults. Autism Research, Oct 2010	Dupl. PM#18
244	Roine, Ulrika; Roine, Timo; Salmi, Juha; et al.: Increased coherence of white matter fiber tract organization in adults with Asperger syndrome: A diffusion tensor imaging study. Autism Research, Dec 2013	Dupl. PM#59
245	Kirkovski, Melissa; Enticott, Peter G.; Maller, Jerome J.; et al. Diffusion tensor imaging reveals no white matter impairments among adults with autism spectrum disorder. Psychiatry Research: Neuroimaging, Jul 30, 2015	Dupl. PM#76
246	Nickel, K.; Tebartz van Elst, L.; Perlov, E.; et al. Altered white matter integrity in adults with autism spectrum disorder and an IQ >100: A diffusion tensor imaging study. Acta Psychiatrica Scandinavica, Jun 2017	Dupl. PM#103
247	Mohajer, Bahram; Masoudi, Maryam; Ashrafi, Agaah; et al. Structural white matter alterations in male adults with high functioning autism spectrum disorder and concurrent depressive symptoms; a diffusion tensor imaging study. Journal of Affective Disorders, Dec 1, 2019	Dupl. PM#136
**Cinahl**	TI (autism spectrum OR autistic OR ASD OR high-functioning autism OR Asperger OR Rett) AND TI (DTI OR diffusion tensor OR tractography) AND TI (adult OR adults) 21.5.2025 → 1 result	
248	Mohajer, Bahram; Masoudi, Maryam; Ashrafi, Agaah; et al. Structural white matter alterations in male adults with high functioning autism spectrum disorder and concurrent depressive symptoms; a diffusion tensor imaging study. Journal of Affective Disorders, Dec2019	Dupl. PM# 136, PsI 6

**Included**	**26**	
Excluded	222	
	Children	79
	No DTI	54
	Review	26
	No ASD	10
	Unrelated	10
	Unfocused	9
	Lumping	7
	Case report/series	4
	Opinion	3
	Pooled	3
	Post-mortem	3
	Animal	2
	Protocol	2
	Overlap	1
	Retracted	0
	Duplicate	9

Supplementary Table S2. Quality Assessment for Diverse Studies (QuADS) – Scale and scores attributed according to each criterion to each study.

QuADS Criteria	0	1	2	3
1. **Theoretical or conceptual underpinning to the research**	No mention at all.	General reference to broad theories or concepts that frame the study. e.g. key concepts were identified in the introduction section.	Identification of specific theories or concepts that frame the study and how these informed the work undertaken. e.g. key concepts were identified in the introduction section and applied to the study.	Explicit discussion of the theories or concepts that inform the study, with application of the theory or concept evident through the design, materials and outcomes explored. e.g. key concepts were identified in the introduction section and the application apparent in each element of the study design.
**2. Statement of research aim/s**	No mention at all.	Reference to what the sought to achieve embedded within the report but no explicit aims statement.	Aims statement made but may only appear in the abstract or be lacking detail.	Explicit and detailed statement of aim/s in the main body of report.
**3. Clear description of research setting and target population**	No mention at all.	General description of research area but not of the specific research environment e.g. 'in primary care.'	Description of research setting is made but is lacking detail e.g. 'in primary care practices in region [x]'.	Specific description of the research setting and target population of study e.g. 'nurses and doctors from GP practices in [x] part of [x] city in [x] country.'
**4. The study design is appropriate to address the stated research aim/s**	No research aim/s stated or the design is entirely unsuitable e.g. a Y/N item survey for a study seeking to undertake exploratory work of lived experiences. .	The study design can only address some aspects of the stated research aim/s e.g. use of focus groups to capture data regarding the frequency and experience of a disease.	The study design can address the stated research aim/s but there is a more suitable alternative that could have been used or used in addition e.g. addition of a qualitative or quantitative component could strengthen the design.	The study design selected appears to be the most suitable approach to attempt to answer the stated research aim/s.
**5. Appropriate sampling to address the research aim/s**	No mention of the sampling approach.	Evidence of consideration of the sample required e.g. the sample characteristics are described and appear appropriate to address the research aim/s.	Evidence of consideration of sample required to address the aim. e.g. the sample characteristics are described with reference to the aim/s.	Detailed evidence of consideration of the sample required to address the research aim/s. e.g. sample size calculation or discussion of an iterative sampling process with reference to the research aims or the case selected for study.
**6. Rationale for choice of data collection tool/s**	No mention of rationale for data collection tool used.	Very limited explanation for choice of data collection tool/s. e.g. based on availability of tool.	Basic explanation of rationale for choice of data collection tool/s. e.g. based on use in a prior similar study.	Detailed explanation of rationale for choice of data collection tool/s. e.g. relevance to the study aim/s, co- designed with the target population or assessments of tool quality.
**7. The format and content of data collection tool is appropriate to address the stated research aim/s**	No research aim/s stated and/or data collection tool not detailed.	Structure and/or content of tool/s suitable to address some aspects of the research aim/s or to address the aim/s superficially e.g. single item response that is very general or an open-response item to capture content which requires probing.	Structure and/or content of tool/s allow for data to be gathered broadly addressing the stated aim/s but could benefit from refinement. e.g. the framing of survey or interview questions are too broad or focused to one element of the research aim/s.	Structure and content of tool/s allow for detailed data to be gathered around all relevant issues required to address the stated research aim/s.
**8. Description of data collection procedure**	No mention of the data collection procedure.	Basic and brief outline of data collection procedure e.g. 'using a questionnaire distributed to staff'.	States each stage of data collection procedure but with limited detail or states some stages in detail but omits others e.g. the recruitment process is mentioned but lacks important details.	Detailed description of each stage of the data collection procedure, including when, where and how data was gathered such that the procedure could be replicated.
**9. Recruitment data provided**	No mention of recruitment data.	Minimal and basic recruitment data, e.g. number of people invited who agreed to take part.	Some recruitment data but not a complete account e.g. number of people who were invited and agreed.	Complete data allowing for full picture of recruitment outcomes e.g. number of people approached, recruited, and who completed with attrition data explained where relevant.
**10. Justification for analytic method selected**	No mention of the rationale for the analytic method chosen.	Very limited justification for choice of analytic method selected. e.g. previous use by the research team.	Basic justification for choice of analytic method selected e.g. method used in prior similar research.	Detailed justification for choice of analytic method selected e.g. relevance to the study aim/s or comment around of the strengths of the method selected.
**11. The method of analysis was appropriate to answer the research aim/s**	No mention at all.	Method of analysis can only address the research aim/s basically or broadly.	Method of analysis can address the research aim/s but there is a more suitable alternative that could have been used or used in addition to offer a	Method of analysis selected is the most suitable approach to attempt answer the research aim/s in detail e.g. for qualitative interpretative

			stronger analysis.	phenomenological analysis might be considered preferable for experiences vs. content analysis to elicit frequency of occurrence of events.
**12. Evidence that the research stakeholders have been considered in research design or conduct.**	No mention at all.	Consideration of some the research stakeholders e.g. use of pilot study with target sample but no stakeholder involvement in planning stages of study design.	Evidence of stakeholder input informing the research. e.g. use of pilot study with feedback influencing the study design/conduct or reference to a project reference group established to guide the research.	Substantial consultation with stakeholders identifiable in planning of study design and in preliminary work e.g. consultation in the conceptualisation of the research, a project advisory group or evidence of stakeholder input informing the work.
**13. Strengths and limitations critically discussed**	No mention at all.	Very limited mention of strengths and limitations with omissions of many key issues. e.g. one or two strengths/limitations mentioned with limited detail.	Discussion of some of the key strengths and weaknesses of the study but not complete. e.g. several strengths/limitations explored but with notable omissions or lack of depth of explanation.	Thorough discussion of strengths and limitations of all aspects of study including design, methods, data collection tools, sample & analytic approach.

**Study – Criterion**	**1**	**2**	**3**	**4**	**5**	**6**	**7**	**8**	**9**	**10**	**11**	**12**	**13**
Bloemen et al., 2010 [67]	2	3	3	2	2	3	2	3	2	3	3	3	3
Thomas et al., 2011 [68]	3	3	3	3	2	2	2	3	3	2	3	2	3
Bakhtiari et al., 2012 [69]	2	3	2	3	2	3	3	2	3	2	2	2	3
Kleinhans et al., 2012 [70]	3	3	3	2	3	3	2	3	3	3	2	3	3
Mueller et al., 2013 [71]	2	2	2	2	3	3	2	3	2	2	3	3	3
Roine et al., 2013 [72]	2	3	2	3	3	2	2	3	2	3	3	2	3
Peeva et al., 2013 [73]	2	3	2	3	3	3	3	3	2	3	3	2	2
Itahashi et al. 2015 [59]	3	2	3	2	2	3	2	2	2	2	3	2	3
Libero et al., 2015 [60]	3	2	3	2	3	2	3	3	2	3	3	3	3
Kirkovski et al., 2015 [74]	3	2	3	3	3	2	2	3	2	3	3	2	3
Ecker et al., 2016 [75]	2	3	2	2	3	2	3	2	3	3	2	3	2
Libero et al., 2016 [76]	3	3	3	2	3	2	3	3	3	3	3	3	3
Nickel et al., 2017 [77]	3	3	3	2	3	3	3	3	2	3	3	2	3
Yamagata et al., 2018 [78]	3	2	3	3	3	2	2	3	2	3	2	3	3
Hattori et al., 2019 [79]	3	2	3	2	2	3	3	3	2	3	3	2	3
Yassin et al., 2019 [80]	3	3	3	2	3	3	3	3	2	3	3	2	3
Mohajer et al., 2019 [81]	3	2	2	2	3	2	3	3	3	3	2	3	3
Haigh et al., 2020 [82]	3	3	3	2	3	3	2	2	3	3	3	2	3
Ohta et al., 2020 [83]	3	2	3	2	3	3	2	3	3	2	3	2	3
Bletsch et al., 2020 [84]	2	3	3	2	3	3	3	2	2	3	3	2	3
Arunachalam Chandran et al., 2021 [85]	2	2	3	2	2	2	2	2	2	2	3	2	2
Yoshikawa et al., 2022 [86]	3	2	3	3	2	3	3	2	3	2	3	3	3
Cai et al., 2022 [87]	3	3	3	2	3	3	2	3	3	2	3	2	3
DiPiero et al., 2023 [88]	3	3	3	3	3	3	2	3	3	2	3	3	3
Weerasekera et al., 2024 [89]	3	2	3	3	2	3	3	3	2	3	3	2	3
Shin et al., 2024 [90]	3	3	3	2	3	3	3	3	3	3	3	3	3

PRISMA

Supplementary Table S3 PRISMA 2020 Checklist

Section and Topic	Item #	Checklist item	Location where item is reported
**TITLE**			1
Title	1	Identify the report as a systematic review.	1
**ABSTRACT**			1
Abstract	2	See the PRISMA 2020 for Abstracts checklist.	1
**INTRODUCTION**			2-3
Rationale	3	Describe the rationale for the review in the context of existing knowledge.	2-3
Objectives	4	Provide an explicit statement of the objective(s) or question(s) the review addresses.	3
**METHODS**			4-5
Eligibility criteria	5	Specify the inclusion and exclusion criteria for the review and how studies were grouped for the syntheses.	4
Information sources	6	Specify all databases, registers, websites, organisations, reference lists and other sources searched or consulted to identify studies. Specify the date when each source was last searched or consulted.	4
Search strategy	7	Present the full search strategies for all databases, registers and websites, including any filters and limits used.	4
Selection process	8	Specify the methods used to decide whether a study met the inclusion criteria of the review, including how many reviewers screened each record and each report retrieved, whether they worked independently, and if applicable, details of automation tools used in the process.	4-5
Data collection process	9	Specify the methods used to collect data from reports, including how many reviewers collected data from each report, whether they worked independently, any processes for obtaining or confirming data from study investigators, and if applicable, details of automation tools used in the process.	4-5
Data items	10a	List and define all outcomes for which data were sought. Specify whether all results that were compatible with each outcome domain in each study were sought (e.g. for all measures, time points, analyses), and if not, the methods used to decide which results to collect.	5
	10b	List and define all other variables for which data were sought (e.g. participant and intervention characteristics, funding sources). Describe any assumptions made about any missing or unclear information.	4-5
Study risk of bias assessment	11	Specify the methods used to assess risk of bias in the included studies, including details of the tool(s) used, how many reviewers assessed each study and whether they worked independently, and if applicable, details of automation tools used in the process.	5
Effect measures	12	Specify for each outcome the effect measure(s) (e.g. risk ratio, mean difference) used in the synthesis or presentation of results.	N/A
Synthesis methods	13a	Describe the processes used to decide which studies were eligible for each synthesis (e.g. tabulating the study intervention characteristics and comparing against the planned groups for each synthesis (item #5)).	5
	13b	Describe any methods required to prepare the data for presentation or synthesis, such as handling of missing summary statistics, or data conversions.	N/A
	13c	Describe any methods used to tabulate or visually display results of individual studies and syntheses.	4-5
	13d	Describe any methods used to synthesize results and provide a rationale for the choice(s). If meta-analysis was performed, describe the model(s), method(s) to identify the presence and extent of statistical heterogeneity, and software package(s) used.	N/A
	13e	Describe any methods used to explore possible causes of heterogeneity among study results (e.g. subgroup analysis, meta-regression).	N/A
	13f	Describe any sensitivity analyses conducted to assess robustness of the synthesized results.	N/A
Reporting bias assessment	14	Describe any methods used to assess risk of bias due to missing results in a synthesis (arising from reporting biases).	5
Certainty assessment	15	Describe any methods used to assess certainty (or confidence) in the body of evidence for an outcome.	5
**RESULTS**			5-20
Study selection	16a	Describe the results of the search and selection process, from the number of records identified in the search to the number of studies included in the review, ideally using a flow diagram.	
	16b	Cite studies that might appear to meet the inclusion criteria, but which were excluded, and explain why they were excluded.	Suppl. T 1
Study characteristics	17	Cite each included study and present its characteristics.	5-20
Risk of bias in studies	18	Present assessments of risk of bias for each included study.	Suppl. T2
Results of individual studies	19	For all outcomes, present, for each study: (a) summary statistics for each group (where appropriate) and (b) an effect estimate and its precision (e.g. confidence/credible interval), ideally using structured tables or plots.	N/A
Results of syntheses	20a	For each synthesis, briefly summarise the characteristics and risk of bias among contributing studies.	Suppl. T2
	20b	Present results of all statistical syntheses conducted. If meta-analysis was done, present for each the summary estimate and its precision (e.g. confidence/credible interval) and measures of statistical heterogeneity. If comparing groups, describe the direction of the effect.	N/A
	20c	Present results of all investigations of possible causes of heterogeneity among study results.	5
	20d	Present results of all sensitivity analyses conducted to assess the robustness of the synthesized results.	N/A
Reporting biases	21	Present assessments of risk of bias due to missing results (arising from reporting biases) for each synthesis assessed.	N/A
Certainty of evidence	22	Present assessments of certainty (or confidence) in the body of evidence for each outcome assessed.	N/A
**DISCUSSION**			20-25

Section and Topic	Item #	Checklist item	Location where item is reported
Discussion	23a	Provide a general interpretation of the results in the context of other evidence.	23
	23b	Discuss any limitations of the evidence included in the review.	23-24
	23c	Discuss any limitations of the review processes used.	23-24
	23d	Discuss implications of the results for practice, policy, and future research.	24-25
**OTHER INFORMATION**			25-26
Registration and protocol	24a	Provide registration information for the review, including register name and registration number, or state that the review was not registered.	5
	24b	Indicate where the review protocol can be accessed, or state that a protocol was not prepared.	5
	24c	Describe and explain any amendments to information provided at registration or in the protocol.	5
Support	25	Describe sources of financial or non-financial support for the review, and the role of the funders or sponsors in the review.	N/A
Competing interests	26	Declare any competing interests of review authors.	23
Availability of data, code and other materials	27	Report which of the following are publicly available and where they can be found: template data collection forms; data extracted from included studies; data used for all analyses; analytic code; any other materials used in the review.	N/A
